# *Colletotrichum fructicola* co-opts cytotoxic ribonucleases that antagonize host competitive microorganisms to promote infection

**DOI:** 10.1128/mbio.01053-24

**Published:** 2024-07-02

**Authors:** Chunhao Wang, Mengqing Han, Yanyan Min, Jiayi Hu, Yuemin Pan, Lili Huang, Jiajun Nie

**Affiliations:** 1 Anhui Province Key Laboratory of Crop Integrated Pest Management, Anhui Agricultural University, Hefei, China; 2 State Key Laboratory for Crop Stress Resistance and High-Efficiency Production, Northwest A&F University, Yangling, Shaanxi, China; Universidade de Sao Paulo, Ribeirao Preto, Sao Paulo, Brazil

**Keywords:** ribonuclease, antimicrobial activity, cell death, *Colletotrichum*, host-associated microorganisms

## Abstract

Phytopathogens secrete numerous molecules into the environment to establish a microbial niche and facilitate host infection. The phytopathogenic fungus *Colletotrichum fructicola,* which causes pear anthracnose, can colonize different plant tissues like leaves and fruits, which are occupied by a diversity of microbes. We speculate that this fungus produces antimicrobial effectors to outcompete host-associated competitive microorganisms. Herein, we identified two secreted ribonucleases, CfRibo1 and CfRibo2, from the *C. fructicola* secretome. The two ribonucleases both possess ribonuclease activity and showed cytotoxicity in *Nicotianan benthamiana* without triggering immunity in an enzymatic activity-dependent manner. CfRibo1 and CfRibo2 recombinant proteins exhibited toxicity against *Escherichia coli*, *Saccharomyces cerevisiae*, and, importantly, the phyllosphere microorganisms isolated from the pear host. Among these isolated microbial strains, *Bacillus altitudinis* is a pathogenic bacterium causing pear soft rot. Strikingly, CfRibo1 and CfRibo2 were found to directly antagonize *B. altitudinis* to facilitate *C. fructicola* infection. More importantly, CfRibo1 and CfRibo2 functioned as essential virulence factors of *C. fructicola* in the presence of host-associated microorganisms. Further analysis revealed these two ribonucleases are widely distributed in fungi and are undergoing purifying selection. Our results provide the first evidence of antimicrobial effectors in *Colletotrichum* fungi and extend the functional diversity of fungal ribonucleases in plant-pest-environment interactions.

**IMPORTANCE:**

*Colletotrichum fructicola* is emerging as a devastating pathogenic fungus causing anthracnose in various crops in agriculture, and understanding how this fungus establishes successful infection is of great significance for anthracnose disease management. Fungi are known to produce secreted effectors as weapons to promote virulence. Considerable progress has been made in elucidating how effectors manipulate plant immunity; however, their importance in modulating environmental microbes is frequently neglected. The present study identified two secreted ribonucleases, CfRibo1 and CfRibo2, as antimicrobial effectors of *C. fructicola*. These two proteins both possess toxicity to pear phyllosphere microorganisms, and they efficiently antagonize competitive microbes to facilitate the infection of pear hosts. This study represents the first evidence of antimicrobial effectors in *Colletotrichum* fungi, and we consider that CfRibo1 and CfRibo2 could be targeted for anthracnose disease management in diverse crops in the future.

## INTRODUCTION

Phytopathogenic microbes are known to secrete numerous effector proteins to interfere with host innate immunity and physiology, thereby promoting disease establishment (1, 2). Notably, each plant has an intricate interaction with its surrounding microbes that constitute different microbial communities. Some of these microbes are adaptive in the same plant, while some serve as beneficial microorganisms for plants. Therefore, beyond host manipulation, a successful phytopathogen must meanwhile outcompete its microbial co-inhabitants for the limited nutrients and niche space, conceivably by effector proteins (3, 4). Recently, several studies have highlighted the roles of antimicrobial effectors in shaping host microbiome (5–7). For instance, VdAve1, a small cysteine-rich protein secreted by the pathogenic fungus *Verticillium dahliae*, exhibits antimicrobial activity and selectively suppresses plant-associated bacteria, facilitating fungal colonization of host plants (7). Similarly, VmAMP2 and VdAMP3, another two antimicrobial effector proteins identified from *V. dahliae*, are capable of inhibiting host-associated bacteria and fungi, respectively (6, 7).

Fungal secretory ribonucleases (RNases) have been reported to serve as effectors that play versatile roles in multi-dimensional plant-pest-environment interactions (8–10). Notably, fungal ribotoxins represent an outstanding class among the members of this family for their cytotoxicity (9). A few RNases play roles in plant immunity modulation. For example, the cytotoxic VdRTX1 from *V. dahliae* and Fg12 from *Fusarium graminearum* are able to trigger immune responses and disease resistance in plants (11, 12). By contrast, CSEP0064/BEC1054, a RNase-like effector from *Blumeria graminis,* suppresses plant immunity (13). Some fungal RNases function as virulence factors, such as the just-mentioned Fg12 and CSEP0064/BEC1054. Similarly, BbRNT2 and BbTrv are required for the virulence of the insect pathogen *Beauveria bassiana* (14). Some RNases are even considered to participate in nutrient uptake due to the finding that Nuc1 and Nuc2 from the smut fungus *Ustilago maydis* utilize host apoplastic RNA as a nutrient (15). Remarkably, recent evidence illustrates that fungal RNases can function as antimicrobial effectors as well. One of the first supporting examples is found in Zt6 from *Zymoseptoria tritici* (8). Zt6 displays toxicity to both bacteria and yeasts but not the pathogen *per se*, and it was supposed to play a role in microbial competition. Most recently, Ribo1 from smut fungi was demonstrated to antagonize two biocontrol bacteria isolated from host leaves, corroborating the roles of fungal RNases in microbiome modulation (16). Despite these findings, the functional diversity of fungal RNases seems not fully discovered. Particularly, considering a certain plant can be infected by multiple pathogens, it remains unknown whether fungal RNases can directly modulate other pathogenic microbes to promote infection.

The genus *Colletotrichum* ranges among the top 10 fungal pathogens in phytopathology (17), among which *C. fructicola* is a pathogenic fungus causing the devastating anthracnose of diverse crops, particularly fruits and vegetables (18). *C. fructicola* is able to colonize diverse host tissues, including flowers, twigs, leaves, and fruits. Taking pear as an example, this fungu*s* can infect both leaves and fruits, resulting in early defoliation and fruit bitter rot, respectively (19). Given the abundant microorganisms on different tissues, this fungus has to cope with a variety of microbial cohabitants during host infection, either being pathogenic, nonpathogenic, or host beneficial. It is tempting to speculate that *C. fructicola* deploys versatile antimicrobial effectors to compete against other microorganisms. However, hitherto only limited effectors have been reported in *C. fructicola* (20–22), with no antimicrobial effectors characterized.

In this study, we analyzed the secretome of *C. fructicola*, leading to the identification of two secreted RNases, which we designated CfRibo1 and CfRibo2, respectively. Both of these two proteins exhibit ribonucleolytic activity, and concurrently, they are cytotoxic to *Nicotiana benthamiana* without triggering plant immunity. Strikingly, we found CfRibo1 and CfRibo2 display antimicrobial activity against both lab stains and phyllosphere microbes of pear. A result of particular note is their direct inhibition of a pathogenic bacterium that causes soft rot of pear. Further analysis revealed that the two RNases play essential roles in fungal virulence in the presence of host phyllosphere microbes. This study represents the first report of antimicrobial effectors from *C. fructicola*, and our data expand the functional diversity of fungal RNases. We also suggest that CfRibo1 and CfRibo2 represent two promising targets for the management of anthracnose disease.

## RESULTS

### Secretome analysis of *C. fructicola* identified two secreted ribonucleases with cytotoxic activity

**Fig 1:**
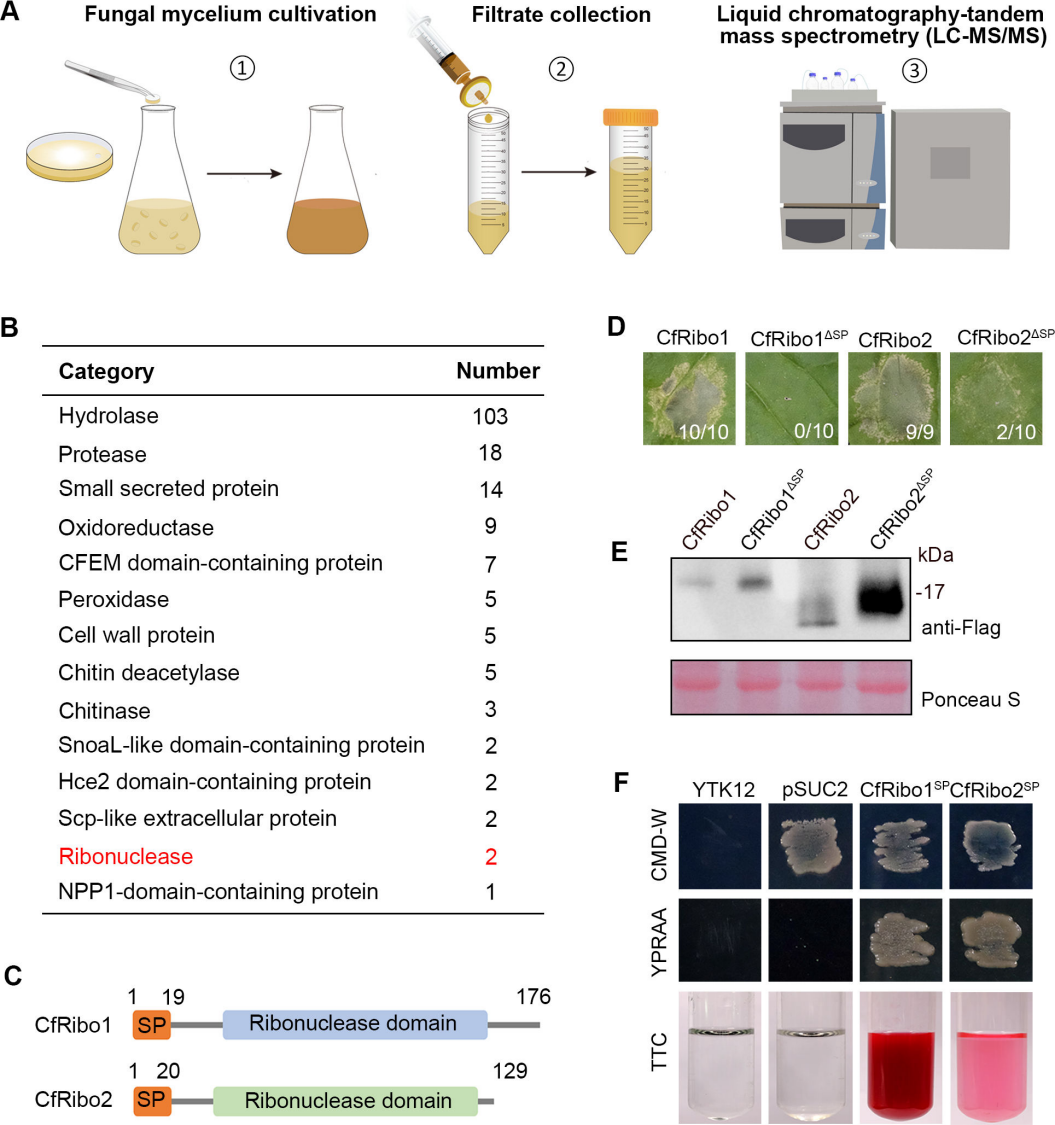
CfRibo1 and CfRibo2 are two cytotoxic ribonucleases identified in *Colletotrichum fructicola* secretome. (**A**) Schematic representation of *C. fructicola* secretome analysis. (**B**) Classification of proteins identified in *C. fructicola* secretome. The numbers of each protein family are shown. (**C**) Schematic diagram illustration of the sequence structure of CfRibo1 and CfRibo2. (**D**) *Nicotiana benthamiana* leaves showing cell death triggered by CfRibo1, CfRibo2, and their signal peptide-deletion versions (CfRibo1^ΔSP^ and CfRibo2^ΔSP^). The proteins were transiently expressed in *N. benthamiana* leaves via agroinfiltration. Cell death was observed 3–5 days post-agroinfiltration, with representative leaves photographed. The number of leaves showing cell death phenotype (numerator) and the number of total surveyed leaves (denominator) are indicated at the bottom of each photographed leaf. (**E**) Immunoblotting analysis of the transiently expressed proteins with anti-Flag antibody. Total proteins were stained with Ponceau S to serve as loading controls. (**F**) Functional validation of the SP of CfRibo1 and CfRibo2 using a yeast secretion system. The empty pSUC2 vector was used as a negative control.

To gain insights into *C. fructicola*-secreted effector proteins, the *C. fructicola* strain DSCF-02 was cultivated by shaking in potato dextrose broth. After 5 days, the culture filtrate was harvested and subjected to liquid chromatography–tandem mass spectrometry analysis (Fig. 1A). In total, 178 proteins were identified in the secretome (Table S1), with hydrolases annotated being the most abundant ones (Fig. 1B). Notably, two ribonucleases were among these identified proteins. Since we focused on potential antimicrobial effectors, these two ribonucleases, which we designated CfRibo1 (XP_031889721.1) and CfRibo2 (XP_031876463.1), respectively, were chosen for further analysis. Sequence analysis revealed that they both contain a predicted N-terminal signal peptide (SP) and a ribonuclease domain in their mature region (Fig. 1C). Considering the cytotoxic activity of many reported ribonucleases, we tested whether they can similarly induce cell death by transient expression in the model plant *N. benthamiana*. As a result, both CfRibo1 and CfRibo2 triggered obvious cell death (Fig. 1D). In contrast, CfRibo1 without SP (CfRibo1^ΔSP^) completely failed to elicit cell death, and CfRibo2 without SP (CfRibo2^ΔSP^)-triggered cell death was greatly compromised compared to that of the full-length version, indicating both ribonucleases require SP for full cytotoxic activity. Immunoblot analysis revealed all proteins were properly expressed (Fig. 1E). To functionally validate the SP of the two proteins, a yeast secretion trap assay and a 2,3,5-triphenyltetrazolium chloride (TTC)-based color reaction assay were performed (23). The yeasts transformed with the SP of CfRibo1 (CfRibo1^SP^) and CfRibo2 (CfRibo2^SP^), but not the empty pSUC2 vector, showed normal growth on selective media (Fig. 1F), suggesting both SPs enabled invertase secretion. A red color can be observed in TTC solution mixed with CfRibo1^SP^- and CfRibo2^SP^-transformed yeasts, confirming the SP-guided invertase secretion. Collectively, these results demonstrate that CfRibo1 and CfRibo2 represent two secreted cytotoxic ribonucleases.

### CfRibo1- and CfRibo2-triggered cell death is not associated with plant immunity activation in *N. benthamiana*

**Fig 2:**
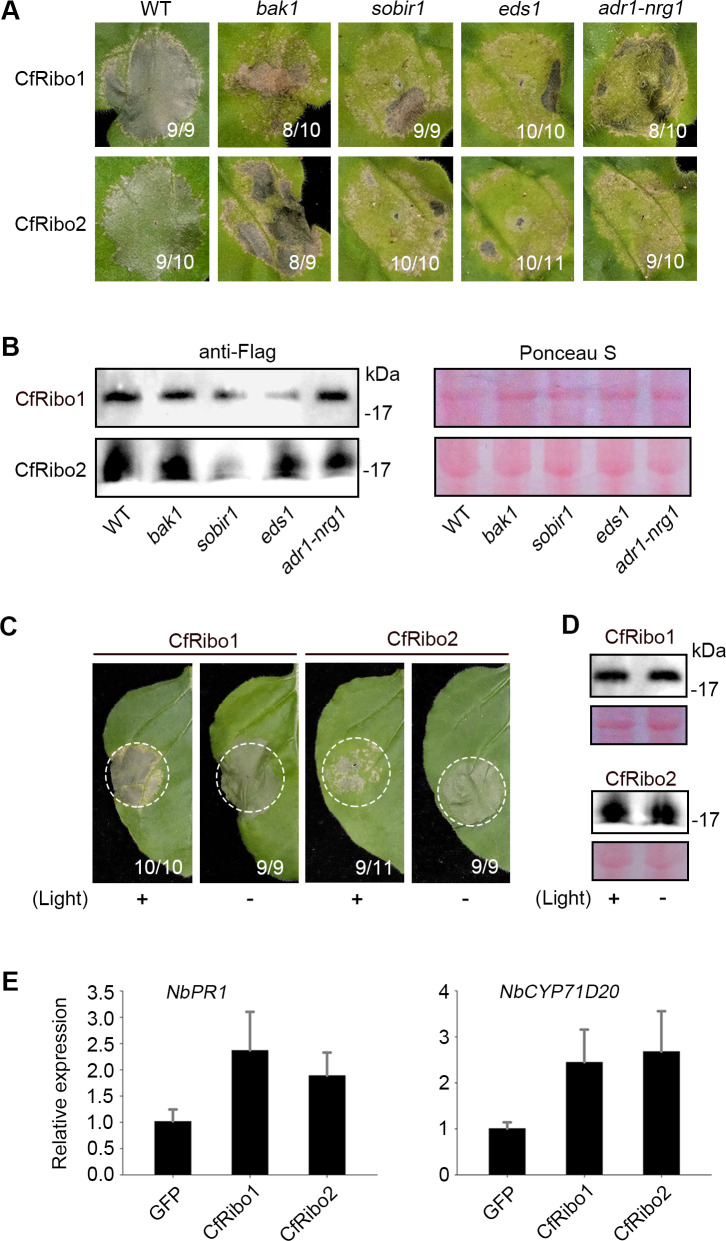
CfRibo1 and CfRibo2 do not activate immunity in *N. benthamiana*. (**A**) Phenotype of cell death triggered by CfRibo1 and CfRibo2 in the wild-type *N. benthamiana* and mutants of central immunity regulators. CfRibo1 and CfRibo2 were agroinfiltrated in the wild-type *N. benthamiana* as well as the CRISPR/Cas9-edited *bak1*, *sobir1*, *eds1*, and *adr1-nrg1* mutants. Cell death was visualized and photographed at 3–5 days post-agroinfiltration (dpa). The number of leaves showing cell death phenotype (numerator) and the number of total surveyed leaves (denominator) are indicated at the bottom of the photographed leaves. (**B**) Immunoblotting analysis of CfRibo1 and CfRibo2 transiently expressed in *N. benthamiana* with anti-Flag antibody. Ponceau S-stained total proteins were shown as loading controls. (**C**) Light independence of CfRibo1- and CfRibo2-triggered cell death. CfRibo1 and CfRibo2 were transiently expressed in the wild-type *N. benthamiana*, followed by incubation under light or dark conditions. Representative leaves showing the phenotypes were photographed at 3–5 dpa. The number of leaves showing cell death phenotype (numerator) and the number of total surveyed leaves (denominator) are indicated at the bottom of the photographed leaves. (**D**) Immunoblotting analysis of transiently expressed CfRibo1 and CfRibo2 with anti-Flag antibody. Total proteins were stained with Ponceau S to serve as loading controls. (**E**) Relative expression analysis of *NbPR1* and *NbCYP71D20* after the transient expression of CfRibo1 and CfRibo2. CfRibo1, CfRibo2, and the GFP control were agroinfiltrated into *N. benthamiana* Leaves. Leaves expressing indicated proteins were sampled at 2 days post-agroinfiltration, and transcripts of *NbPR1* and *NbCYP71D20* were evaluated by reverse transcription-quantitative polymerase chain reaction.

To determine whether CfRibo1 and CfRibo2 are involved in plant immunity activation, we first used CRISPR/Cas9-edited *N. benthamiana* mutants including *bak1* (24), *sobir1* (25), *eds1* (26), and the double mutant *adr1-nrg1* (27) for analysis. Transient expression assays revealed that CfRibo1 and CfRibo2 triggered normal cell death in all tested mutants (Fig. 2A), and immunoblot analysis showed the proteins were normally expressed (Fig. 2B). The independence of these immune regulators suggests that CfRibo1 and CfRibo2 are probably not involved in plant perception and immunity activation. As some fungal RNases rely on light for cell death activation, we then assayed whether CfRibo1 and CfRibo2 function in a similar way. As was shown, both of them retained full cell death-inducing activity in *N. benthamiana* under darkness (Fig. 2C), with protein accumulations unaltered (Fig. 2D), demonstrating the cytotoxicity of CfRibo1 and CfRibo2 is light independent. Finally, we tested whether plant immune-related genes like *PR1* (28) and *CYP71D20* (29) can be activated. In accordance with the above results, the transcript levels of both tested genes were not significantly changed in *N. benthamiana* leaves transiently expressing either CfRibo1 or CfRibo2, compared to that of the GFP control (Fig. 2E). Therefore, despite their cytotoxic activity, CfRibo1 and CfRibo2 do not serve as immune elicitors.

### Ribonucleolytic activity of CfRibo1 and CfRibo2 determines cytotoxicity

**Fig 3:**
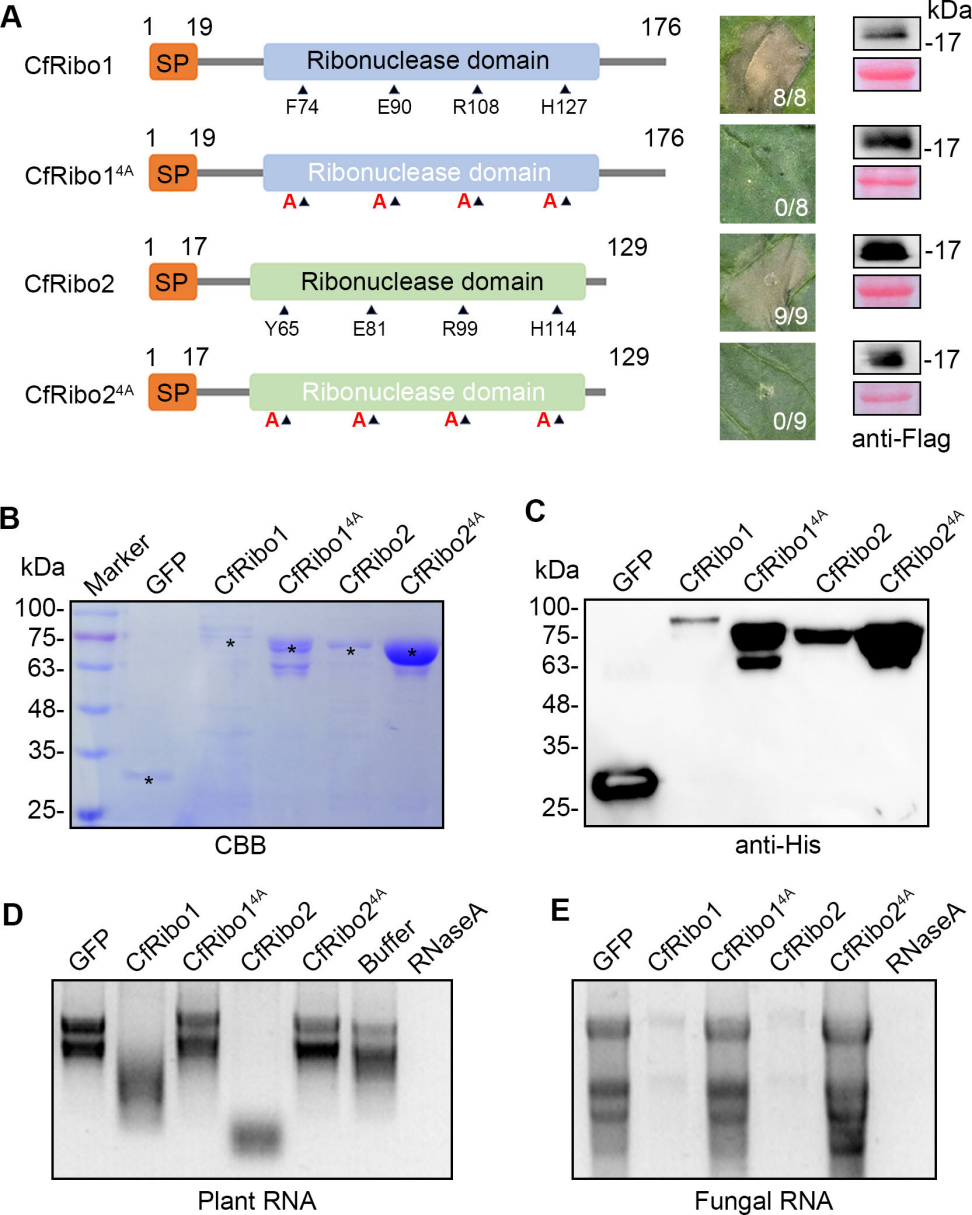
Ribonucleolytic activity of CfRibo1 and CfRibo2 determines their cytotoxicity. (**A**) Cell death triggered by CfRibo1, CfRibo2, and their catalytic site-mutated versions CfRibo1^4A^ and CfRibo2^4A^ in *N. benthamiana*. Schematic diagram illustration of these proteins was indicated. Each protein was transiently expressed in *N. benthamiana* via agroinfiltration. Cell death was observed at 3–5 days post-agroinfiltration, with representative leaves photographed. The number of leaves showing cell death phenotype (numerator) and the number of total surveyed leaves (denominator) are indicated at the bottom of the photographed leaves. The expression of these proteins was confirmed by immunoblotting with anti-Flag antibody, and the Ponceau S-stained total proteins were shown as loading controls. (**B**) Detection of recombinant proteins on gels by Coomassie brilliant blue staining. GFP, CfRibo1, CfRibo2, CfRibo1^4A^, and CfRibo2^4A^ recombinant proteins produced in *Escherichia coli* were subjected to sodium dodecyl sulfate-polyacrylamide gel electrophoresis. The protein bands of expected size were indicated with black asterisks. (**C**) Immunoblotting detection of the recombinant proteins with anti-His antibody. (**D and E**) Ribonucleolytic activity tests using the recombinant proteins. The proteins were incubated with either plant total RNA or fungal total RNA at 37°C for 30 min. GFP recombinant protein and buffer were used as negative controls, and RNase A was used as a positive control.

The ribonuclease domain of CfRibo1 and CfRibo2 each contains four catalytic sites (Fig. 3A). To assess whether the catalytic sites are responsible for cytotoxic activity, the four residues among their ribonuclease domain were substituted with alanine to generate CfRibo1^4A^ and CfRibo2^4A^, respectively. Intriguingly, each mutated version completely lost cell death-inducing activity after transient expression in *N. benthamiana*, and all proteins were normally accumulated by immunoblotting analysis (Fig. 3A). As a control, the cytotoxicity of the commercial RNase A was also tested in *N. benthamiana*, with an obvious cell death-inducing activity observed (Fig. S1). These results suggest that the ribonucleolytic activity of the two proteins is most likely required for their cytotoxicity. As a way to validate this speculation, we tried to obtain the recombinant proteins in *Escherichia coli* and test their RNase activity. Coomassie brilliant blue‐stained gels indicated that the protein abundance of both CfRibo1 and Ribo2 was relatively low (Fig. 3B). By contrast, the abundance of CfRibo1^4A^ and CfRibo2^4A^ was much higher than that of wild-type versions. Western blotting analysis confirmed this result (Fig. 3C). The observed abundance difference probably attributed to the cytotoxicity of CfRibo1 and CfRibo2 in bacterial cells. We next evaluated the ribonucleolytic activity of CfRibo1 and CfRibo2 recombinant proteins by incubation with RNA, with the commercial RNase A as a positive control. We found both CfRibo1 and CfRibo2 degraded total plant RNA (Fig. 3D). In contrast, CfRibo1^4A^, CfRibo2^4A^, GFP, and the buffer solution for recombinant proteins all failed to degrade RNA. Similarly, CfRibo1 and CfRibo2 but not CfRibo1^4A^ or CfRibo2^4A^ degraded total fungal RNA as well (Fig. 3E). Altogether, it can be concluded that CfRibo1 and CfRibo2 possess ribonucleolytic activity that is intimately associated with their cytotoxicity.

### Deletion of CfRibo1 and CfRibo2 has no effect on pathogen virulence during normal inoculations

**Fig 4:**
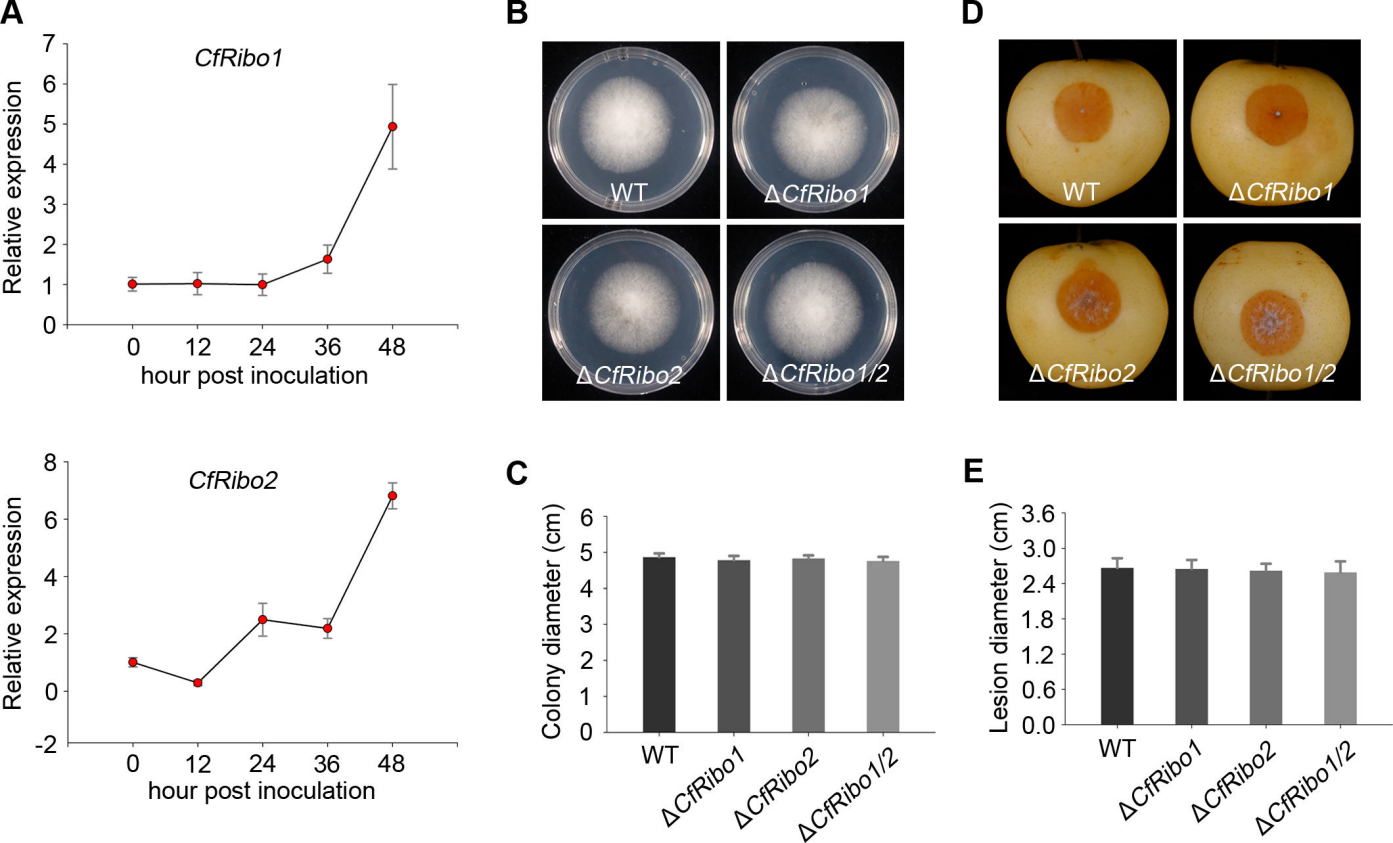
Deletion of *CfRibo1* and *CfRibo2* does not affect *C. fructicola* virulence. (**A**) Expression profiles of *CfRibo1* and *CRibo2* during *C. fructicola* infection of the pear host. Pear fruits inoculated with *C. fructicola* were sampled at 0, 12, 24, 36, and 48 h post-inoculation, respectively. Relative expression of *CfRibo1* and *CfRibo2* was determined by reverse transcription-quantitative polymerase chain reaction. *CfActin* was used as an internal reference to normalize gene expression. (**B and C**) Filamentous growth phenotype of *C. fructicola* and the gene deletion transformants. The wild-type (WT) *C. fructicola* DSCF-02, Δ*CfRibo1*, Δ*CfRibo2*, and the double deletion mutant Δ*CfRibo1/2* were cultured on potato dextrose agar plates at 28°C. Photographs were taken, and colony diameter was calculated 3 days later. (**D and E**) Virulence phenotype of *C. fructicola* and the gene deletion transformants. Conidia of *C. fructicola* strains and the gene deletion transformants were inoculated in pear fruits. Disease lesions were photographed, and lesion diameters were calculated 3 days post-inoculation.

To explore the role of CfRibo1 and CfRibo2 in pathogen virulence, we first analyzed their expression profiles during *C. fructicola* infection of the pear host. As was shown, the transcripts of *CfRibo1* and *CfRibo2* were significantly induced from 36-h post-inoculation (hpi) to 48 hpi (Fig. 4A), suggesting the two RNases possibly contribute to *C. fructicola* virulence at late stages of infection.

To further investigate their virulence roles, we generated deletion mutants for *CfRibo1* (Δ*CfRibo1*) and *CfRibo2* (Δ*CfRibo2*) (Fig. S2). To eliminate their potential functional redundancy, we also obtained double deletion mutants (Δ*CfRibo1/2*) by knocking out both of them. All mutants and the wild type displayed a similar colony phenotype on potato dextrose agar (PDA) plates (Fig. 4B and C), suggesting CfRibo1 and CfRibo2 do not affect pathogen filamentous growth. After inoculation in the pear fruit, disease lesions caused by these mutants were also comparable to that of the wild type (Fig. 4D and E). Therefore, CfRibo1 and CfRibo2 do not appear to be genetically linked to pathogen virulence. We additionally tested whether the two RNases participate in pathogen tolerance to environmental abiotic stresses like oxidative stress, salt, membrane damage, cell wall integrity, and osmotic pressure. By cultivation on PDA supplemented with corresponding chemicals, we likewise observed no apparent difference in colony phenotype between the wild type and the deletion mutants (Fig. S3).

### CfRibo1 and CfRibo2 possess antimicrobial activity against host-associated microorganisms

**Fig 5:**
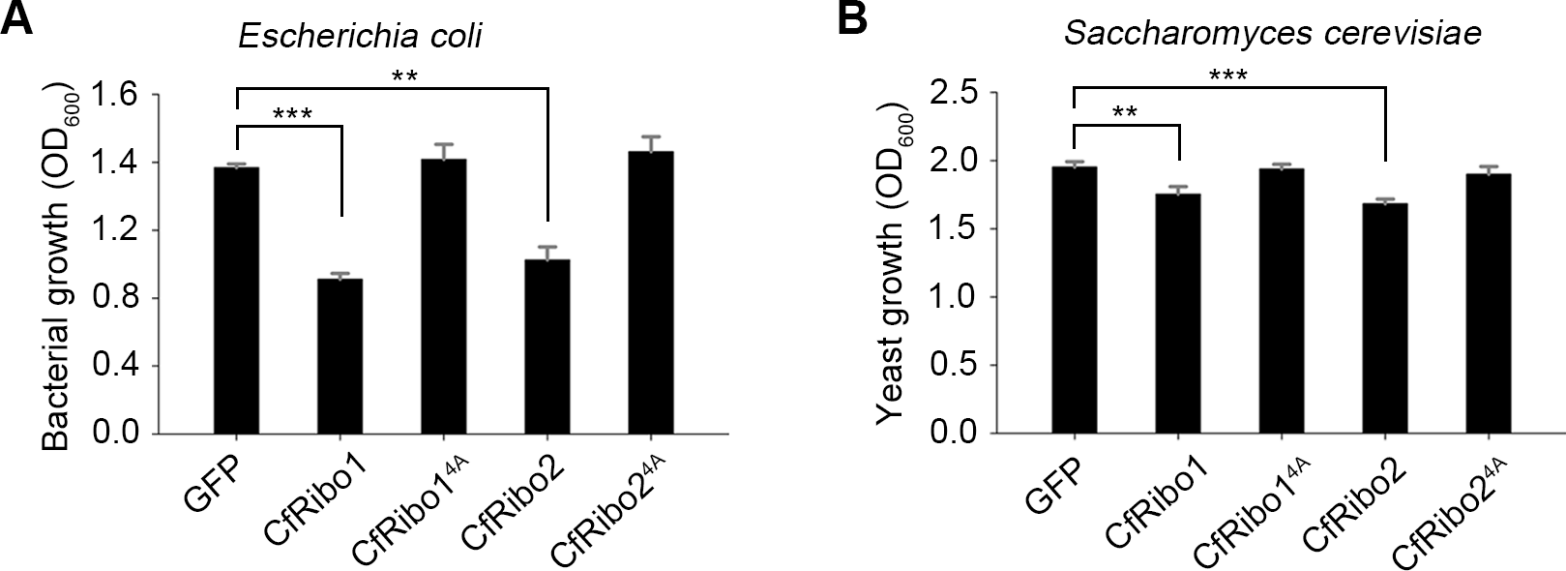
CfRibo1 and CfRibo2 exhibit antimicrobial activity against *E. coli* and *Saccharomyces cerevisiae*. GFP, CfRibo1, CfRibo2, CfRibo1^4A^, and CfRibo2^4A^ recombinant proteins were incubated with *E. coli* and *S. cerevisiae* at 1 µM concentration, followed by culturing at 37°C and 30°C, respectively. Both bacterial growth (**A**) and yeast growth (**B**) were monitored 24 h later. Error bars indicate the mean ± SD (Student’s *t*-test, **P < 0.01 and ***P < 0.001).

Based on the above results, the exact biological roles of CfRibo1 and CfRibo2 still remain a question. Considering the antimicrobial activity of several reported RNases (8, 16), we then asked whether CfRibo1 and CfRibo2 function as antimicrobial effectors. Before testing this hypothesis, we first assayed the antimicrobial activity of *C. fructicola* culture filtrate containing the secreted proteins and found that it significantly inhibited the growth of the prokaryotic *Escherichia coli* and the eukaryotic *Saccharomyces cerevisiae* (Fig. S4). Then, *in vitro* microbial toxicity assays were performed with the recombinant proteins. As a result, CfRibo1 and CfRibo2 exhibited potent toxicity to *E. coli*, whereas CfRibo1^4A^ and CfRibo2^4A^ did not (Fig. 5A). This result was consistent with the poor expression of both CfRibo1 and CfRibo2 in *E. coli* (Fig. 2B). Similarly, the two RNases also displayed toxicity to *S. cerevisiae* (Fig. 5B). Intriguingly, CfRibo1 and CfRibo2 showed no toxicity to *C. fructicola* and even promoted the mycelium development of this fungus (Fig. S5A). Following this up, exogenous treatment with CfRibo1 and CfRibo2 facilitated the infection of *C. fructicola* in pear fruits (Fig. S5B and C).

**Fig 6:**
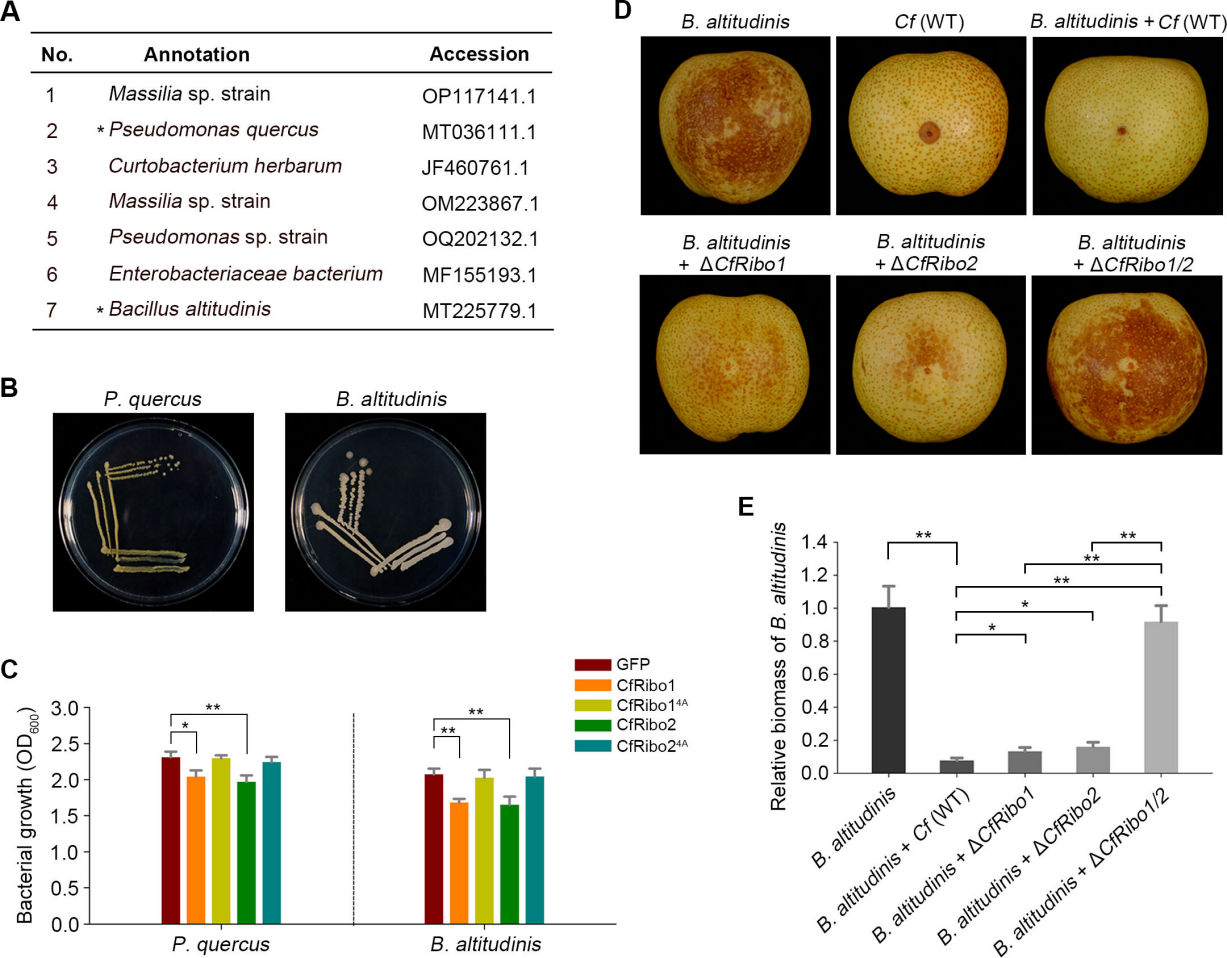
CfRibo1 and CfRibo2 synergistically antagonize a pathogenic bacterium during the infection of pear fruits. (**A**) Seven bacterial strains were identified from pear leaves. *Pseudomonas quercus* and *Bacillus altitudinis* chosen for further analysis were marked with black asterisks. (**B**) Colony phenotype of *P. quercus* and *B. altitudinis* on lysogeny broth plates. (**C**) Antimicrobial activity of CfRibo1 and CfRibo2 against *P. quercus* and *B. altitudinis*. GFP, CfRibo1, CfRibo2, CfRibo1^4A^, and CfRibo2^4A^ recombinant proteins were incubated with *P. quercus* and *B. altitudinis* at 1 µM concentration, followed by culturing at 37°C. The growth of each strain was monitored 24 h post-incubation. Error bars indicate the mean ± SD (Student’s *t*-test, **P < 0.01). (**D and E**) CfRibo1 and CfRibo2 synergistically antagonize *B. altitudinis* during infection. *C. fructicola* strains including the wild type [*Cf* (WT)] and the gene deletion transformants (Δ*CfRibo1*, Δ*CfRibo2*, and Δ*CfRibo1/2*) were co-inoculated with *B. altitudinis* in pear fruits. As controls, *Cf* (WT) and *B. altitudinis* were individually inoculated at the same time. Disease symptoms were photographed at 40 hpi. Infection of *B. altitudinis* was quantified by relative biomass using reverse transcription-quantitative polymerase chain reaction. *CfActin* from *C. fructicola* and *PbrTubulin* from pear were used as reference genes. Error bars indicate the mean ± SD (Student’s *t*-test, *P < 0.05 and **P < 0.01).

Because the tested *E. coli* and *S. cerevisiae* are lab strains, it remains unclear whether CfRibo1 and CfRibo2 also possess toxicity to host-associated microorganisms, particularly the pathogenic microbes. To answer this question, we isolated the phyllosphere microorganisms from pears. As the two RNases exhibited more potent antimicrobial activity against the prokaryotic *E. coli* (Fig. 5A and B), host-associated bacteria were mainly focused on. Consequently, a total of seven bacterial isolates were obtained after determination by 16S rDNA sequencing (Fig. 6A). Among these, *Pseudomonas quercus* was reported to be associated with leaf spot disease of *Quercus mongolica* (30), whereas *Bacillus altitudinis* serves as a causal agent of soft rot in pears and apples (31), for which these two strains were mainly focused on for further analysis (Fig. 6B). Microbial toxicity assays revealed that the growth of these two strains was obviously inhibited by CfRibo1 and CfRibo2 (Fig. 6C). Additionally, CfRibo1 suppressed the growth of the other five isolated bacterial strains, while CfRibo2 antagonized three of them (No. 1, 4, and 5) (Fig. S6). Therefore, CfRibo1 and CfRibo2 also exhibit antimicrobial activity against host-associated microorganisms.

### CfRibo1 and CfRibo2 synergistically antagonize a pathogenic bacterium during host infection

Inoculation assays showed that *P. quercus* was unable to cause any disease symptoms in pear fruit, while *B. altitudinis* caused severe pear rot (Fig. S7), demonstrating *B. altitudinis* indeed functions as a pathogenic bacterium of pear. To ascertain whether CfRibo1 and CfRibo2 can antagonize *B. altitudinis* during infection, the deletion mutants and *B. altitudinis* were co-inoculated on pear. Much to our surprise, the wild-type *C. fructicola* almost completely suppressed *B. altitudinis* infection, as no visible rot symptom was observed in the pear inoculated with both pathogens (Fig. 6D). Notably, deletion of either *CfRibo1* or *CfRibo2* partially restored bacterial infection, while deletion of both genes enabled the full virulence of *B. altitudinis*. To confirm this result, the relative biomass of *B. altitudinis* was further measured, with similar results obtained (Fig. 6E). These data strongly suggest that CfRibo1 and CfRibo2 play vital roles in antagonizing other pathogenic microbe(s) during host infection. It has to be mentioned that *B. altitudinis* also greatly inhibited the infection of *C. fructicola*, as the fungal lesions were greatly compromised in the presence of this bacterium (Fig. 6D), suggesting *C. fructicola* and *B. altitudinis* represent potent competitors during infection of their common host. We further tested *C. fructicola*’s response to the other isolated bacteria and found three additional strains (No. 1, 2, and 3), including *P. quercus*, also suppressed *C. fructicola* infection (Fig. S8), implying multiple microbial co-inhabitants serve as competitors of *C. fructicola*.

### CfRibo1 and CfRibo2 are essential for fungal virulence in the presence of niche competitor(s)

**Fig 7:**
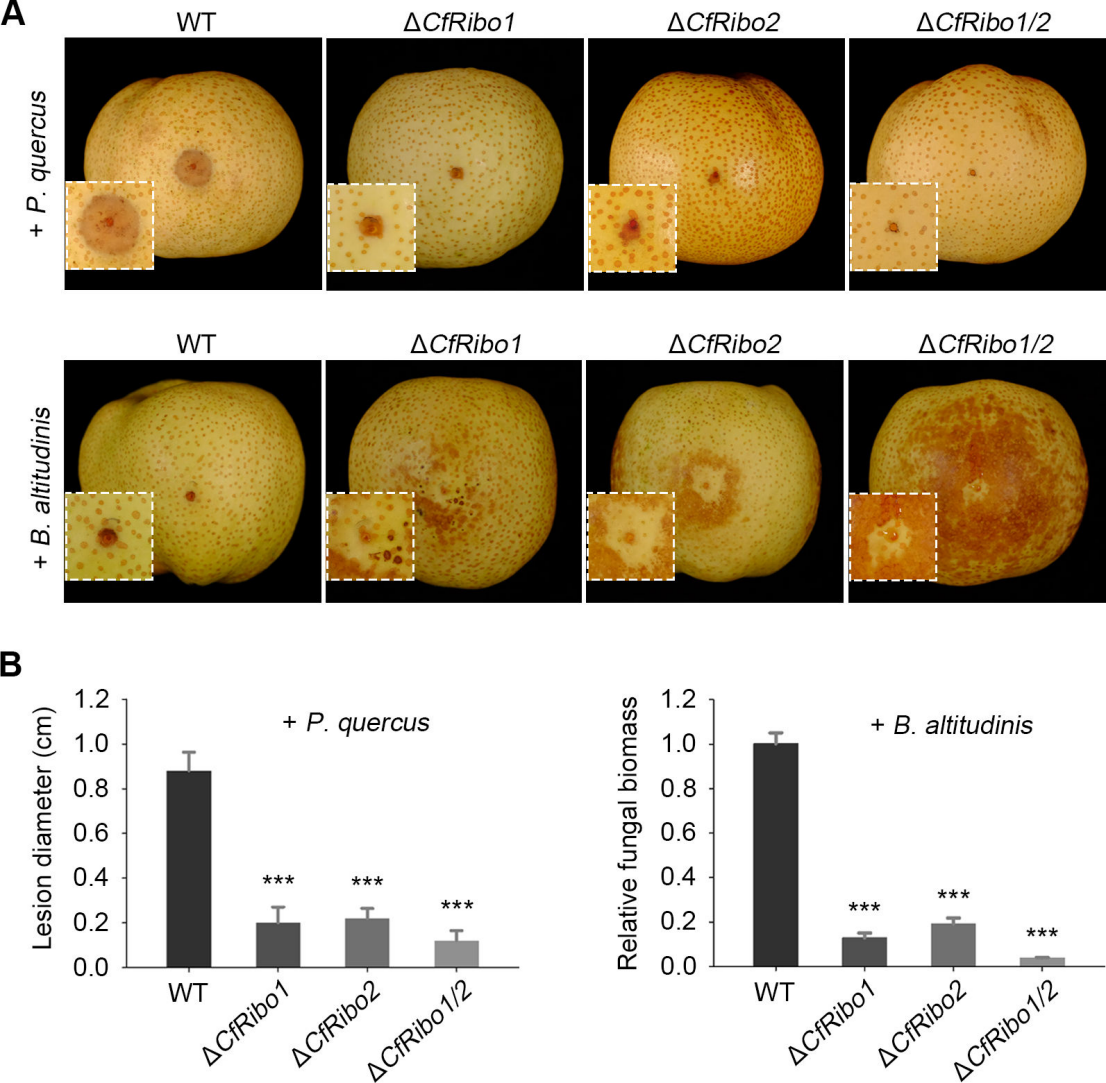
CfRibo1 and CfRibo2 play essential roles in fungal virulence in the presence of *P. quercus* and *B. altitudinis*. (**A**) Virulence phenotype of the wild-type *C. fructicola* and gene deletion transformants in the presence of *P. quercus* and *B. altitudinis*. Conidia of the wild-type DSCF-02 strain and the gene deletion transformants (Δ*CfRibo1*, Δ*CfRibo2*, and Δ*CfRibo1/2*) were co-inoculated with *P. quercus* or *B. altitudinis* in pear fruits. Disease lesions were photographed at 48 hpi. (**B**) Quantification of *C. fructicola* and *B. altitudinis* infections. Disease development of *C. fructicola* was calculated with lesion diameters. *B. altitudinis* infection was determined by reverse transcription-quantitative polymerase chain reaction, with *CfActin* and *PbrTubulin* used as reference genes. Error bars indicate the mean ± SD (Student’s *t*-test, ***P < 0.001).

The antimicrobial activity of CfRibo1 and CfRibo2 prompted us to re-evaluate their virulence role in the presence of host-associated microorganisms. To this end, the deletion mutants were co-inoculated with *P. quercus* and *B. altitudinis*, respectively, and fungal disease development was re-calculated. In the presence of *P. quercus*, we noticed greatly attenuated disease lesions caused by either Δ*CfRibo1*, Δ*CfRibo2*, or Δ*CfRibo1/2* compared to that of the wild type (Fig. 7A and B). In the presence of *B. altitudinis*, we were unable to distinguish the lesions caused by *C. fructicola*; however, relative fungal biomass similarly showed significantly compromised virulence of the deletion mutants. Therefore, CfRibo1 and CfRibo2 function as essential virulence factors in the presence of competitive microorganisms.

**Fig 8:**
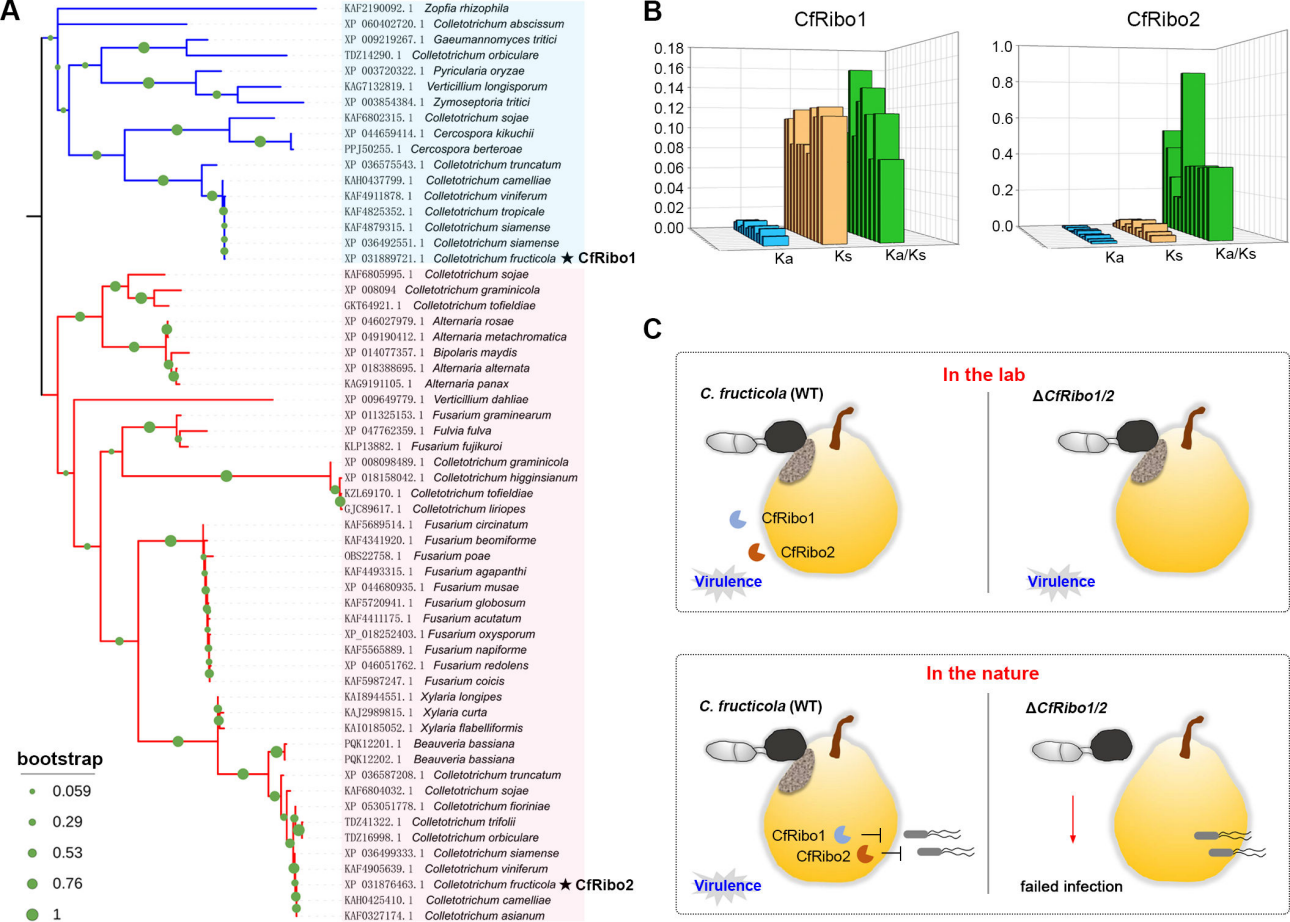
CfRibo1 and CfRibo2 are widely distributed across fungal taxa. (**A**) Phylogeny distribution of CfRibo1 and CfRibo2 homologs from different fungal species. A maximum-likelihood method was adopted to construct the tree. Bootstrap percentage support for each branch is indicated by circle symbols. CfRibo1 and CrRibo2 are marked with black asterisks. (**B**) Selective pressure determination of CfRibo1 and CfRibo2 by Ka/Ks ratios. The nonsynonymous rates (Ka), synonymous rates (Ks), and Ka/Ks ratios of CfRibo1 and CfRibo2 are shown. (**C**) A proposed model for the function of CfRibo1 and CfRibo2 during *C. fructicola*-pear interactions. In the lab, when inoculation assays were performed with *C. fructicola* only, CfRibo1 and CfRibo2 were dispensable for fungal virulence. In nature where diverse microorganisms exist, CfRibo1 and CfRibo2 function as antimicrobial effectors that synergistically antagonize host-associated competitive microorganism(s) and execute essential virulence roles, thereby promoting host infection.

We next investigated the phylogeny distribution of CfRibo1 and CfRibo2. BLAST searches identified an array of homologs for them. Phylogenetic analysis revealed that CfRibo1 and CfRibo2 are widely spread in fungal species, in particular, the genus *Colletotrichum* (Fig. 8A). Sequence alignment showed both CfRibo1 and CfRibo2 are highly conserved among *Colletotrichum* fungi (Fig. S9). Furthermore, the Ka/Ks ratios were calculated to determine the selective pressure of the two RNases. As shown in Fig. 8B, the Ka/Ks ratios of both proteins were less than 1, suggesting CfRibo1 and CfRibo2 are subjected to purifying selection. Taken together, our data demonstrate the secreted RNases CfRibo1 and CfRibo2 antagonize host-associated competitor(s) in nature, thereby controlling *C. fructicola* virulence for host niche protection (Fig. 8C). Promisingly, the two RNases can be targeted for anthracnose disease management.

## DISCUSSION

Quite a few species located in the genus *Colletotrichum* are economically important pathogens causing anthracnose in diverse plants (32). *C. fructicola* is a pathogenic fungus with a wide range of hosts, in particular, horticultural crops (18). *C. fructicola* colonizes different host tissues during its lifecycle, for which it has to compete with diverse surrounding microorganisms to facilitate infection. Herein, we identified two cytotoxic RNases, CfRibo1 and CfRibo2, as antimicrobial effectors of *C. fructicola*. These two effectors synergistically function as essential virulence factors in the presence of host-associated microorganisms. Remarkably, CfRibo1 and CfRibo2 are indispensable for competing with *B. altitudinis*, a pathogenic bacteria of pear host.

A total of 178 proteins were identified from the *C. fructicola* secretome, though these are absolutely not all secreted proteins of this fungus. A majority of the annotated proteins like hydrolases, proteases, chitinases, and chitin deacetylases frequently serve as fungal effectors in plant-microbe interactions (33–36). Among these proteins, two secreted RNases, which we designated CfRibo1 and CfRibo2, were focused on. They both possess cell death-inducing activity after transient expression in *N. benthamiana*, indicating their cytotoxicity similar to that of other reported ribotoxins. Some cytotoxic RNases serve as plant immunity elicitors, such as VdRTX1 and Fg12 from *V. dahliae* and *F. graminearum*, respectively (11, 12). However, in contrast to the plant immunity-inducing activity of VdRTX1 and Fg12, both CfRibo1 and CfRibo2 failed to trigger immune responses when transiently expressed in *N. benthamiana* (Fig. 2). Cell death-inducing proteins produced by fungi frequently function as immune elicitors recognized by plants (37). In the plant immune system, the receptor-like kinases, BAK1 and SOBIR1, the nucleocytoplasmic lipase-like protein, EDS1, as well as the functional redundant helper NLRs, ADR1 and NRG1, are essential immune regulators (29, 38–40). We found CfRibo1- and CfRibo2-triggered cell death was independent of these components, substantiating that CfRibo1 and CfRibo2 do not serve as plant immunity elicitors. This is reminiscent of necrosis and ethylene-inducing peptide 1-like proteins (NLPs) produced by diverse microbes, which have been demonstrated to play versatile roles in cytotoxicity and plant immunity activation (41). Quite a few NLPs exhibit cytotoxicity without activating immune responses in plants, while several of them, such as PyolNLP5 and PyolNLP7 from *Pythium oligandrum,* can simultaneously trigger plant immunity and disease resistance (42). It is unknown whether CfRibo1 and CfRibo2 are able to trigger ROS burst and other hallmarks of immune responses; however, our data strongly indicate CfRibo1 and CfRibo2 are not recognized by plants despite their cytotoxicity. Fungal RNase-triggered cell death has been well documented to result from their cleavage of the sarcin-ricin loop of ribosomal RNA and the subsequent inhibition of protein synthesis (9, 43). Likewise, we found CfRibo1 and CfRibo2 recombinant proteins exhibit RNase activity (Fig. 3). CfRibo1- and CfRibo2-triggered cell death was shown to be dependent on RNase activity, suggesting their cytotoxicity is closely linked to enzymatic activity, similar to the case of Fg12 (11). Notably, a recent study illustrated that the ribonuclease UhRibo1 from *Ustilago hordei* triggers plant cell death independent of its RNase activity (16). Therefore, it appears that fungal RNases display intricate diversification in plant immunity manipulation as well as the connection between cytotoxicity and enzymatic activity.

A few RNases have been illustrated to be virulence effectors of pathogenic fungi. For instance, FoRnt2 and the above-mentioned Fg12 from *Fusarium oxysporum* and *F. graminearum* are required for full virulence, respectively (11, 44). Despite their cytotoxicity and induced expression during late-stage infection, knocking out *CfRibo1* and *CfRibo2* individually or both of them showed no apparent influence on *C. fructicola* virulence, indicating they are not genetically associated with pathogen virulence (Fig. 4). A possible explanation for this observation is the common virulence redundancy of secreted effectors (45, 46). In addition, we found the deletion mutants did not affect pathogen tolerance to abiotic stresses (Fig. S3). These results puzzled us a lot and prompted us to determine the ultimate biological role(s). Considering the antimicrobial activity of several reported RNases like Zt6 from *Zymoseptoria tritici* and the aforementioned UhRibo1 (8, 16), we determined the potential antimicrobial activity of CfRibo1 and CfRibo2. Both of them displayed cytotoxicity to lab strains like *E. coli* and *S. cerevisiae* in an enzymatic activity-dependent manner (Fig. 5). Moreover, they were also demonstrated to be toxic to phyllosphere bacteria species isolated from pear leaves, including *P. quercus* and *B. altitudinis* (Fig. 6; Fig. S6). Possibly, fungal RNases enter the cells of microbes by binding phospholipids in the membrane to facilitate membrane penetration, similar to the case of *Aspergillus* ribotoxins (9, 47, 48). It is worth noting that *B. altitudinis* is a pathogenic bacterium causing pear soft rot (Fig. S7). Therefore, CfRibo1 and CfRibo2 are likely involved in the interaction between *C. fructicola* and its niche competitors including other pathogenic microbes. In support of this, co-inoculation assays revealed that either CfRibo1 or CfRibo2 considerably suppressed the infection of *B. altitudinis* in pear fruits. Though fungal RNases have been demonstrated to engage in antimicrobial competition, to the best of our knowledge, CfRibo1 or CfRibo2 represent the first reported RNases directly antagonizing other phytopathogens to promote infection.

Even if CfRibo1 and CfRibo2 are cytotoxic to different microbes, they seem to pose no toxicity to *C. fructicola per se*, as supported by two lines of evidence. First, the wild-type *C. fructicola* strain and the deletion mutants exhibited no difference in filamentous growth (Fig. 4B). On the other hand, exogenous application of CfRibo1 or CfRibo2 recombinant proteins did not inhibit mycelial development of *C. fructicola* but conversely facilitated fungal growth (Fig. S5). Consistent with this, exogenous application of the recombinant proteins promoted the infection of *C. fructicola* in an enzymatic activity-dependent manner. This result is similar to the case of Zt6, in which the recombinant Zt6 can partly enhance the propagation of *Z. tritici* (8). Many microbes, especially bacteria, possess toxin-antitoxin systems that enable them to inhibit cell growth by producing specific toxins while counteracting their cognate toxins with antitoxins (49). It remains unknown how fungi protect themselves from their secreted cytotoxic RNases thus far. We have not yet determined whether RNases produced by other microbes are toxic to *C. fructicola*. However, beyond any doubt, the specific toxicity of *C. fructicola* RNases to environmental microorganisms makes them ideal weapons for fungal survival in nature.

Importantly, co-inoculation with the two host phyllosphere bacteria (*P. quercus* and *B. altitudinis*) revealed greatly impaired virulence of C*fRibo1* and *CfRibo2* deletion mutants (Fig. 7), suggesting CfRibo1 and CfRibo2 execute potent virulence functions in the presence of host-associated microbes. This result is reminiscent of UhRibo1 and *Verticillium dahliae* VdAMP3 (6, 16). In this context, UhRibo1 contributes to the virulence of smut fungi when co-inoculated with two biocontrol bacteria isolated from maize. Similarly, VdAMP3, an antimicrobial protein secreted by *V. dahliae*, facilitates microsclerotia formation when other competitive fungi are present. In nature, each plant host is occupied by numerous microorganisms, and lab inoculation assays with only one pathogen actually do not reflect the real infection situation in the wild. For this reason, the virulence role of effectors, in particular, the antimicrobial effectors, could sometimes be misevaluated in the lab. Given the essential virulence role of CfRibo1 and CfRibo2 in co-inoculation assays, they would be targeted for anthracnose disease management. More importantly, these two RNases are well conserved in fungal pathogens, especially the genus *Colletotrichum*, and are undergoing purifying selection (Fig. 8). Such features make CfRibo1 and CfRibo2 promising targets of *Colletotrichum* fungi. Hopefully, agents targeting fungal RNases will be engineered in future.

## MATERIALS AND METHODS

### Plants, strains, and their cultivation

*Nicotiana benthamiana* seedlings were grown in a climate chamber (16 h photoperiod) at 22°C. The *Escherichia coli* strain Top10 used for vector construction and ArcticExpress (DE3) used for recombinant protein expression were cultured on lysogeny broth (LB) medium at 37°C. The *Agrobacterium tumefaciens* strain GV3101 used for transient expression was cultured on LB medium at 28°C. The yeast strain YTK12 used for signal sequence trap was maintained on YPDA medium at 25°C. The wild-type *Colletotrichum fructicola* strain DSCF-02 was cultured on PDA at 28°C.

### Vector construction

CfRibo1- and CfRibo2-encoding sequences were cloned from the cDNA library of *C. fructicola* using Phanta Max Super-Fidelity DNA Polymerase (Vazyme, Nanjing, China), and the amplicons were cloned into the binary vector pCAMBIA1300 for transient expression assays using ClonExpress II One-Step Cloning Kit (Vazyme, Nanjing, China). To generate site-mutated versions (CfRibo1^4A^ and CfRibo2^4A^), synthesized double-strand DNAs produced by GenScript (GenScript Biotech Corporation, China) were used as templates. For recombinant protein expression in *E. coli*, the sequences were cloned into pCold-TF vector digested with specific enzymes, using pCAMBIA1300 vectors as templates. For yeast signal sequence trap, the SP sequences were cloned into pSUC2 vector. The primers used are listed in Table S2.

### Functional validation of signal peptides

The signal peptides of CfRibo1 and CfRibo2 were functionally validated via a yeast secretion system as described (23). In brief, pSUC2 vectors containing SP sequences were mobilized into the yeast strain YTK12. After screening on CMD-W plates, positive colonies were further picked for secretion analysis on YPRAA medium plates (Coolaber, Beijing, China). As the pSUC2 vector contains an SP-truncated invertase, a functional SP can rescue the secretion of invertase, thereby enabling normal growth of the yeast on a selective medium. An additional way to validate these secretory SPs was achieved by a color reaction assay. The yeast culture was incubated with 0.1% 2,3,5-triphenyltetrazolium chloride solution (Coolaber, Beijing, China) at 37°C for 10 min. Secretion of the invertase guided by a functional SP will turn the TTC into an insoluble, red-colored form.

### Transient expression and immunoblotting analysis

pCAMBIA1300 constructs were mobilized into *A. tumefaciens* via electroporation. After screening on selective LB medium, positive colonies were picked for propagation with a shaking incubator. After ~48 h, bacterial cells were collected via centrifugation and washed twice with ultra-distilled water, followed by suspension in infiltration buffer (10 mM MgCl_2_, 10 mM MES, and 200 µM acetosyringone, pH 5.7) for 2 h at room temperature. Prior to infiltration into *N. benthamiana* leaves with a needleless syringe, the suspension was adjusted to an OD_600_ of 0.8 with infiltration buffer. Cell death triggered by transiently expressed proteins was detected 3–5 days post-agroinfiltration.

For immunoblotting analysis, the leaves were sampled 36–48 h post-agroinfiltration. Total proteins were extracted with lysis buffer [50 mM Tris, 150 mM NaCl, 0.5% Triton X-100, 1% proteinase inhibitor cocktail, and 1 mM phenylmethanesulfonyl fluoride (PMSF), pH 7.5]. After boiling in 5× sodium dodecyl sulfate loading buffer, the protein samples were subjected to electrophoresis and were subsequently transferred to a polyvinylidene difluoride membrane (Roche). The membrane was probed with HRP-conjugated anti-Flag antibody (ABclonal, Wuhan, China), and protein bands were detected with an electrochemiluminescence substrate kit (GE Healthcare).

### Protein expression in *E. coli*

The pCold-TF constructs were mobilized into the *E. coli* strain ArcticExpress (DE3), and a positive colony was picked for propagation in the LB medium. Protein expression was induced for 24 h at 16°C in the presence of 0.2 mM isopropyl-β-D-thiogalactopyranoside. Bacterial cells were then harvested by centrifugation and washed twice with PBS buffer. To extract recombinant proteins, harvested cells were transferred into lysis buffer (20 mM Na_2_HPO_4_, 300 mM NaCl, pH 7.4, 1 mg/mL lysozyme, and 1 mM PMSF) for sonication. Afterward, the supernatant containing crude proteins was collected by centrifugation, and the recombinant proteins were purified using Ni NTA Beads (Smart-Lifesciences, Inc., China) following the manufacturer’s instructions. Protein expressions were visualized by Coomassie brilliant blue staining and were further detected by immunoblotting with HRP-conjugated anti-His antibody (ABclonal, Wuhan, China).

### RNA extraction and relative expression analysis

Total RNA from collected samples was isolated by FastPure Cell/Tissue Total RNA Isolation Kit (Vazyme, Nanjing, China), and cDNA was synthesized by HiScript II 1st Strand cDNA Synthesis Kit (Vazyme, Nanjing, China) following the manufacturer’s instructions. Relative expression was performed via reverse transcription-quantitative polymerase chain reaction (RT-qPCR) using Hieff qPCR SYBR Green Master Mix (Yeasen, Shanghai, China). *CfActin* and *NbActin* were chosen as internal references to normalize gene expressions in *C. fructicola* and *N. benthamiana*, respectively. Transcript levels were analyzed through the 2^-ΔΔCT^ method (50).

### RNase activity and antimicrobial activity assays

To test the RNase activity of CfRibo1 and CfRibo2, 1 µM recombinant proteins were separately incubated with 1 µg plant total RNA or fungal total RNA in DEPC-water in an RNase-free 1.5 mL Eppendorf tube. The commercial RNase A (Takara Bio) was used as a positive control. After incubation at 37°C for 30 min, the mixtures were subjected to agarose gel electrophoresis to visualize the degradation of RNA.

To determine the antimicrobial activity of the recombinant proteins, the *E. coli* strain ArcticExpress (DE3) and the *Saccharomyces cerevisiae* strain YTK12 were appropriately cultured in LB liquid medium and YPDA liquid medium, respectively. *C. fructicola* strain DSCF-02 was cultured in potato dextrose broth (PDB) medium to produce conidia. After collection by centrifugation, *E. coli* and *S. cerevisiae* cells were washed twice with ultra-distilled water. Following this, *E. coli* suspension was diluted to a final OD_600_ = 0.1 in LB liquid medium, with *S. cerevisiae* diluted to OD_600_ = 0.5 in YPDA liquid medium. *C. fructicola* conidia were collected by filtering through Miracloth (Millipore) and were adjusted to a concentration of 1 × 10^7^ conidia/mL in 5% PDB. The recombinant proteins were separately mixed with the prepared cells and conidia to a final volume of 3 mL at 1 µM concentration, followed by culturing at 37°C (*E. coli*), 30°C (*S. cerevisiae*), or 28°C (*C. fructicola*). The growth of *E. coli* and *S. cerevisiae* was monitored 24 h later with a UV-visible spectrometer. The growth of *C. fructicola* was monitored and photographed 36 h later with an optical microscope. The antimicrobial activity of *C. fructicola* filtrate against *E. coli* and *S. cerevisiae* was analyzed likewise.

For the determination of antimicrobial activity against host**-**associated bacteria, pear leaves were collected from pear orchards in Dangshan County, China. The leaves were soaked in ultra-distilled water supplemented with 0.05% Tween-20 for 1 h. The washing solution was plated on LB solid medium and incubated at 30°C for 3 days. Individual colonies were cultured and sequenced with 16S rDNA primers to determine their identity. The antimicrobial activity against the isolated bacterial strains including *Pseudomonas quercus* and *Bacillus altitudinis* was tested similar to that of *E. coli*.

### Transformants generation

*C. fructicola* genomic DNA was isolated using FastPure Cell/Tissue DNA Isolation Mini Kit (Vazyme, Nanjing, China). A split-maker strategy was adopted to create gene replacement cassettes (Fig. S10). An upstream flanking sequence (663 bp) and a downstream flanking sequence (673 bp) of *CfRibo1* were cloned from the genomic DNA. Similarly, an upstream flanking sequence (600 bp) and a downstream flanking sequence (578 bp) of *CfRibo2* were cloned. The amplicons for each gene were fused with the geneticin resistance gene (*NEO*) to form replacement cassettes.

Gene knock out transformants were obtained by polyethylene glycol-mediated protoplast transformation. Briefly, six fresh mycelium plugs of *C. fructicola* collected from PDA plates were cultured in PDB for 5 days to produce conidia. An appropriate amount of conidia was cultured in PDB (28°C, 200 rpm, 18–20 h) to produce young mycelia. The young mycelia were harvested and successively washed with ultra-distilled water and 1.2 M KCl, followed by incubation with the lysis buffer [1.2 M MgSO_4_, pH 5.5, 40 mg/mL Vinotaste Pro (Novozymes), and 10 mg/mL Driselase (Sigma-Aldrich)]. After lysis for 3 h (30°C, 110 rpm), protoplasts were filtered through a layer of Miracloth (Millipore) and were harvested by centrifugation (5,000 rpm, 10 min). The harvested protoplasts were washed with STC buffer twice and further diluted to a density of 10^7^ /mL. To perform protoplast transformation, the gene replacement cassettes (10 µg) were pipetted into reaction buffer (200 µL protoplasts, 50 mM spermidine, and 2.5 mg/mL heparin sodium). After incubation for 30 min on ice, 40% PEG8000 was added to the mixture for another 20 min of incubation, following which 10 mL TB3 buffer was used to regenerate the protoplasts. The transformants were selected with geneticin or hygromycin (125 µg/mL) and validated by PCR analysis. For double deletion of *CfRibo1* and *CfRibo2*, the flanking sequences of *CfRibo2* were fused with the hygromycin phosphotransferase gene (*HPH*), and protoplast transformation was likewise performed under the background of Δ*CfRibo1*.

### Pathogen inoculation assays

Conidia of the wild-type *C. fructicola* and its transformants were prepared to a density of 10^7^ /mL. Before pathogen inoculations, pear fruits were decontaminated by wiping with 70% ethanol and washing twice with ultra-distilled water. Each pear fruit was inoculated with 20 µL conidia. The inoculated fruits were placed in trays sealed with plastic wrap to keep high humidity and were placed in a greenhouse (28°C) for disease development. For virulence tests in the presence of host phyllosphere microbes, the bacterial cells were prepared to a concentration of OD_600_ = 0.2. The prepared conidia and bacteria were mixed at a 1:1 ratio before the inoculation of pear fruits. Relative biomass of *C. fructicola* was quantified by RT-qPCR using *CfActin* and *PbrTubulin* as reference genes, and relative bacterial biomass was calculated with 16S rDNA and *PbrTubulin* as references in a similar way.

### Bioinformatics analysis

The signal peptide of the CfRibo1 and CfRibo2 was predicted online through Phobius (https://phobius.sbc.su.se/). Homologs of these two proteins were identified from the NCBI database through BLAST searching programs. Multiple sequence alignment was conducted online through ClustalW (https://www.genome.jp/tools-bin/clustalw). Phylogenetic analysis was performed based on maximum likelihood using MEGA X (51). For selective pressure analysis, nonsynonymous rates (Ka), synonymous rates (Ks), and Ks/KS ratios were calculated using TBtools software (52).

## SUPPLEMENTAL MATERIAL

Supplemental figures mbio.01053-24-s0001.docx Fig. S1 to S10.

Table S1 mbio.01053-24-s0002.xlsx Proteins identified in *C. fructicola* secretome.

Table S2 mbio.01053-24-s0003.xlsx Primers used in this study.

ASM does not own the copyrights to Supplemental Material that may be linked to, or accessed through, an article. The authors have granted ASM a non-exclusive, world-wide license to publish the Supplemental Material files. Please contact the corresponding author directly for reuse.
