# Supplementary material for: Colletotrichum fructicola co-opts cytotoxic ribonucleases that antagonize host competitive microorganisms to promote infection
Source: mBio. 2024 Jul 2;15(8):e01053-24. doi: 10.1128/mbio.01053-24 (PMC11323725; doi:10.1128/mbio.01053-24)
Supplement: Supplemental figures — Fig. S1 to S10. [file mbio.01053-24-s0001.docx]

**
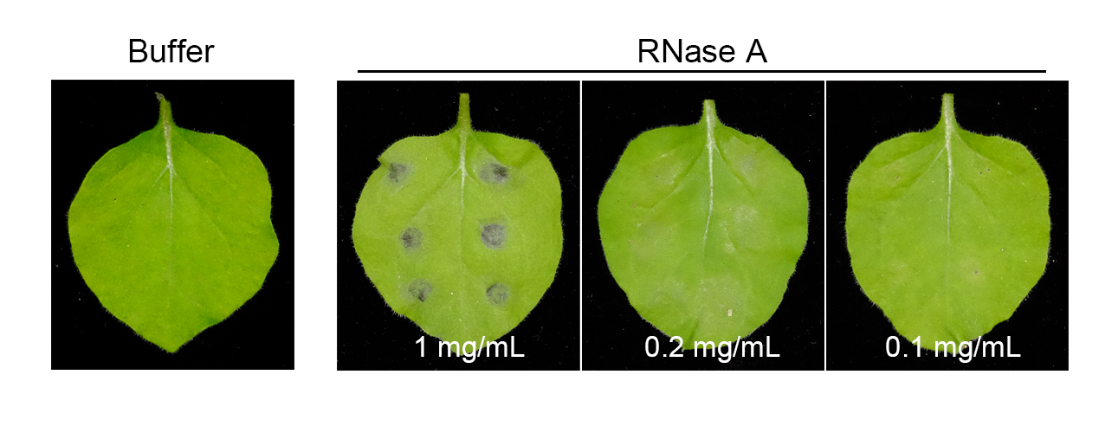
**

**Fig. S1** The commercial RNase A shows cytotoxicity in *Nicotiana benthamiana*. The commercial RNase A was diluted with its dilution buffer to 1 mg/L, 0.2 mg/mL, and 0.1 mg/mL, respectively, and was infiltrated to *N. benthamiana* leaves with a needleless syringe. Dilution buffer was infiltrated as a negative control. Cell death was observed 24 h post infiltration and representative leaves were photographed.

**
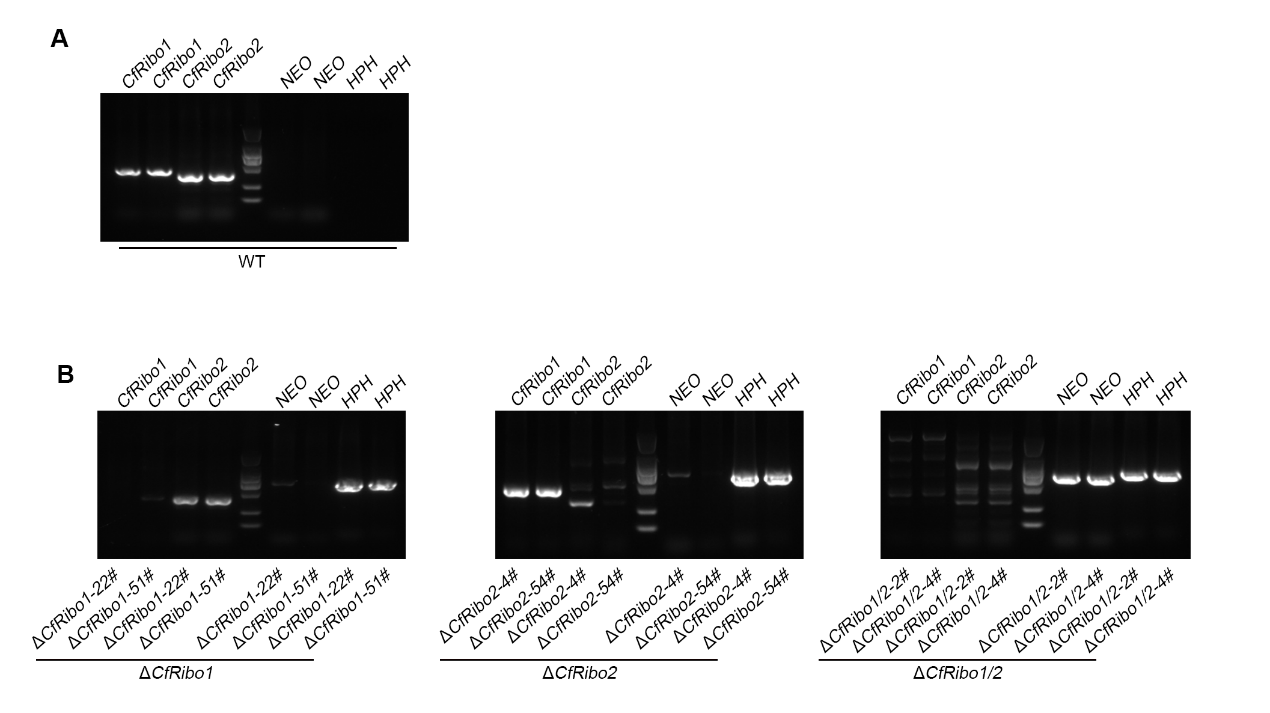
**

**Fig. S2** Validation of *Colletotrichum fructicola* deletion mutants by PCR. The existence of *CfRibo1*, *CfRibo2*, geneticin resistance gene (*NEO*) and hygromycin phosphotransferase gene (*HPH*) was detected with specific primer pairs, with genomic DNA from the wild type *C. fructicola* strain DSCF-02 (A) and the gene deletion transformants (B) used as templates.


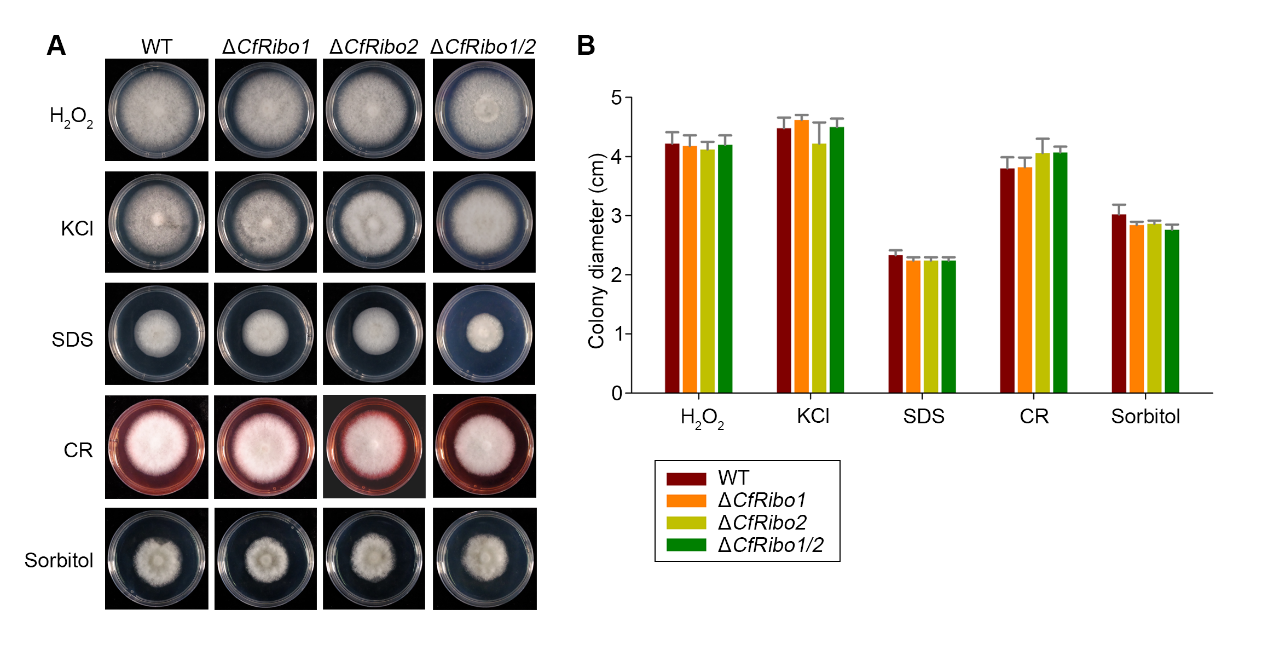


**Fig. S3** Deletion of *CfRibo1* and *CfRibo2* shows no effect on *C. fructicola* tolerance to abiotic stress. The WT and gene deletion transformants of *C. fructicola* were cultured on potato dextrose agar (PDA) plates with 6 mM H_2_O_2_, 0.3 M KCl, 10 % SDS, 200 μg/mL Congo red (CR), and 1 M sorbitol, respectively. Photographs were taken and colony diameters were calculated after cultivation at 28°C for 3 d.


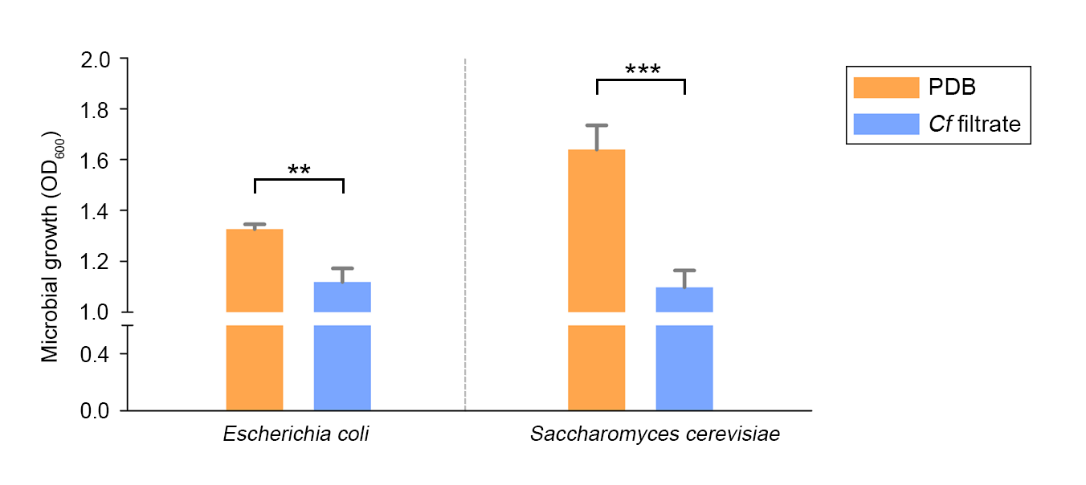


**Fig. S4** *C. fructicola* culture filtrate possesses antimicrobial activity. The culture filtrate (*Cf* filtrate) was incubated with either *Escherichia coli* or *Saccharomyces cerevisiae* at a 1:1 ratio (v/v), followed by culturing at 37°C and 30°C, respectively. The fresh PDB culture was used as a control. The microbial growth was monitored 24 h later. Error bars indicate mean ± SD (Student’s *t*-test, **, *P* < 0.01; ***, *P* < 0.001).

**
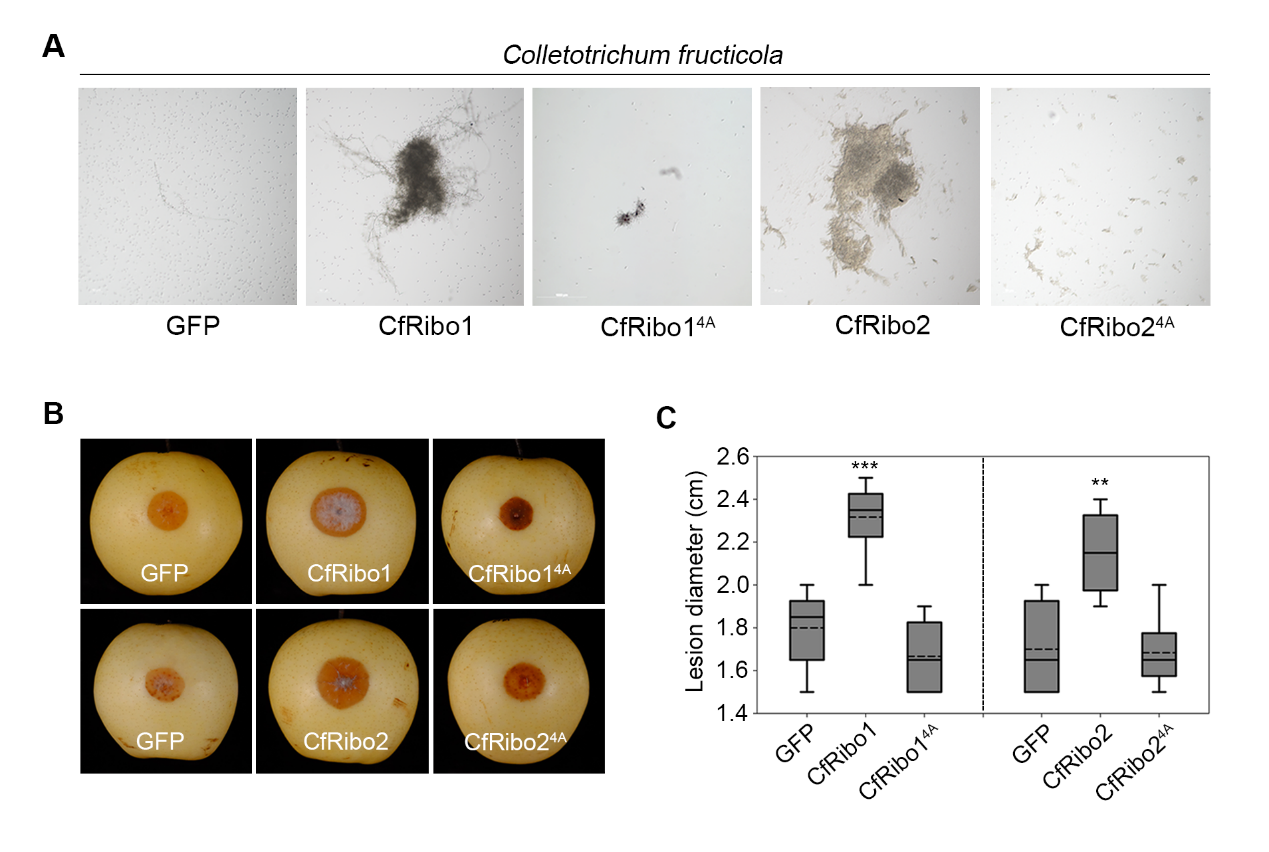
**

**Fig. S5** Exogenous application of CfRibo1 and CfRibo2 recombinant proteins promote *C. fructicola* development and virulence. (A) Phenotype of *C. fructicola* development in the presence of recombinant proteins. GFP, CfRibo1, CfRibo2, CfRibo1^4A^, and CfRibo2^4A^ recombinant proteins produced were incubated with *C. fructicola* conidia in in 5% potato dextrose broth (PDB). Fungal development was monitored and photographed after cultivation at 28°C for 36 h. (B, C) Virulence phenotype of *C. fructicola* in pear fruits in the presence of recombinant proteins. *C. fructicola* conidia were separately mixed GFP, CfRibo1, CfRibo2, CfRibo1^4A^, and CfRibo2^4A^ recombinant proteins, followed by inoculation in pear fruits. Disease lesions were photographed and lesion diameters were calculated 3 d post inoculation. Error bars indicate mean ± SD (Student’s *t*-test, **, *P* < 0.01; ***, *P* < 0.001).


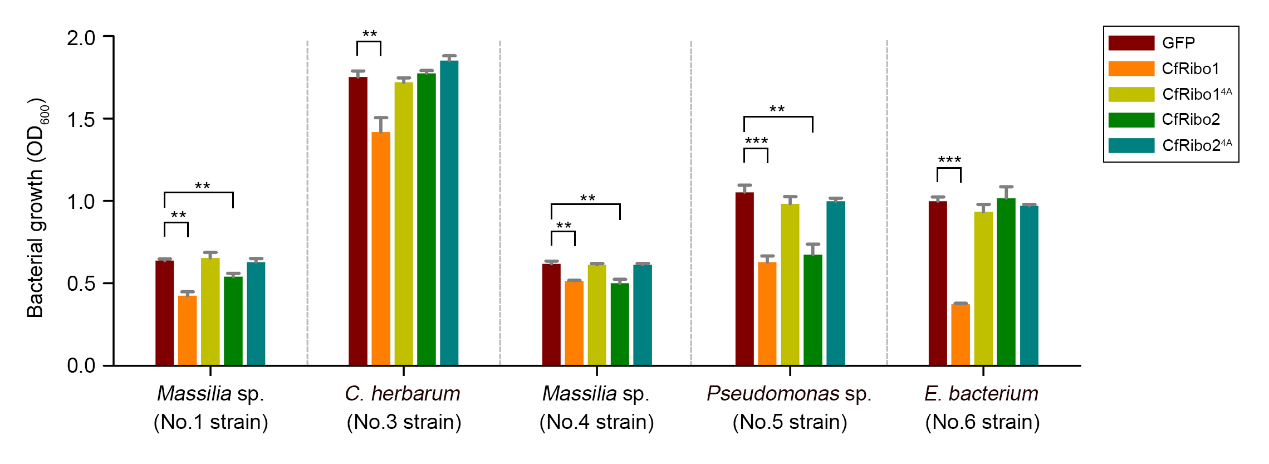


**Fig. S6** CfRibo1 and CfRibo2 differentially antagonize the other five isolated bacteria. GFP, CfRibo1, CfRibo2, CfRibo1^4A^, and CfRibo2^4A^ recombinant proteins were incubated with the five bacteria strains at 1 μM concentration, followed by culturing at 37°C. The growth of each strain was monitored 24 h post incubation. Error bars indicate mean ± SD (Student’s *t*-test, **, *P* < 0.01; ***, *P* < 0.001).


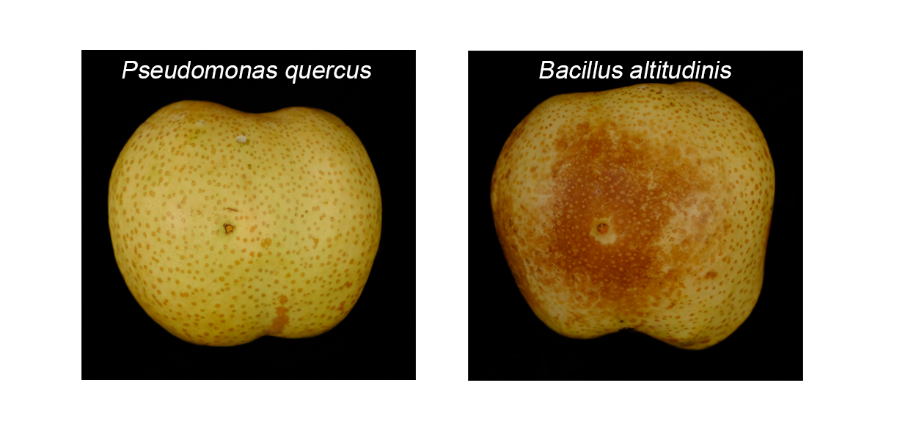


**Fig. S7** *Bacillus altitudinis* is a pathogenic bacterium in pear. *Pseudomonas quercus* and *B. altitudinis* were inoculated in pear fruits. Disease symptom was photographed 48 h post inoculation.


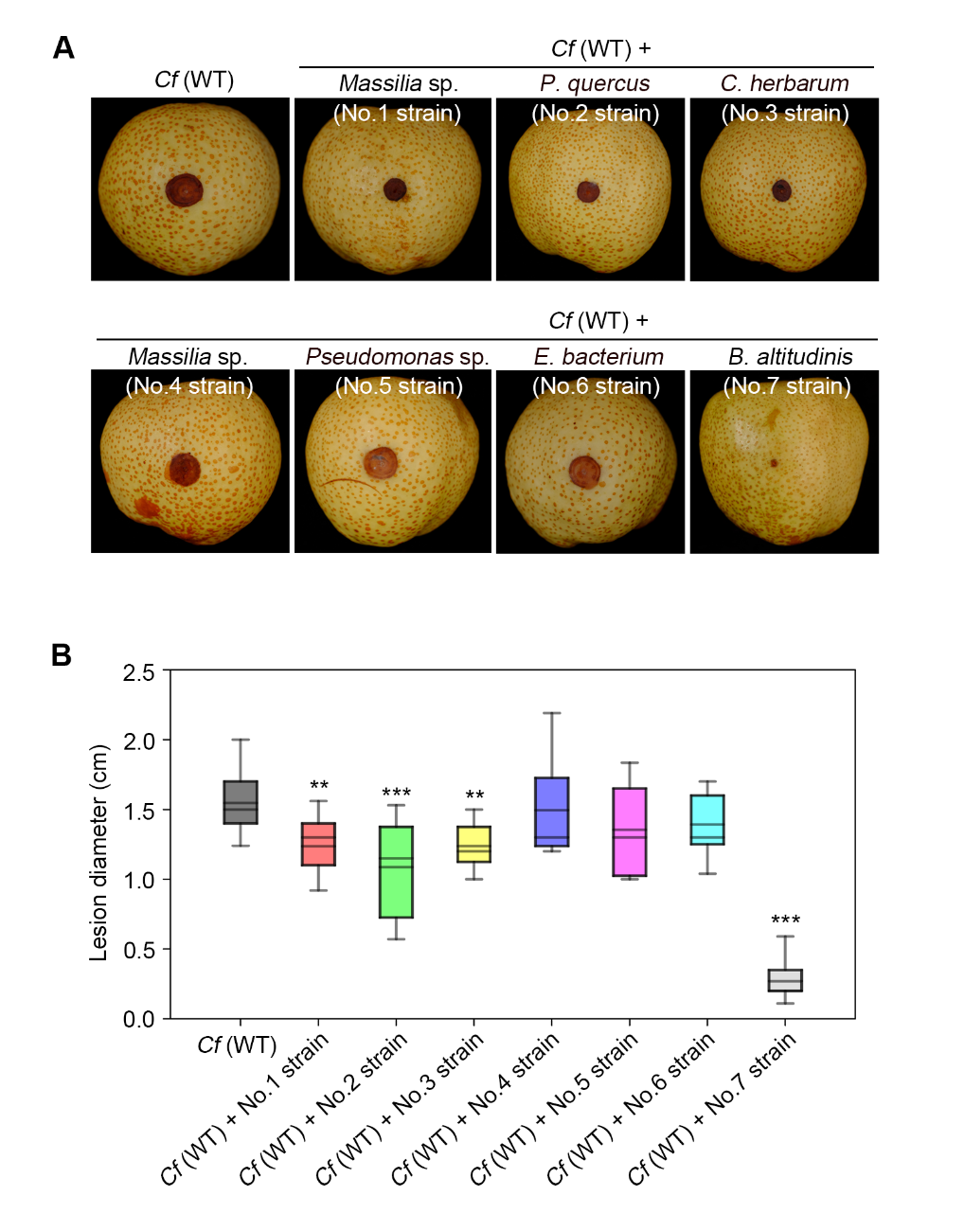


**Fig. S8** Evaluation of *C. fructicola* response to the isolated bacteria strains during pear infection. (A) Virulence phenotype of the wild type *C. fructicola* in the presence of phyllosphere bacterial strains. The wild type *C. fructicola* strain DSCF-02 (*Cf* (WT)) was separately co-inoculated with the seven isolated bacteria in pear fruits. The wild type alone was inoculated as a control. Disease symptoms were monitored and photographed 48 hpi. (B) Disease development calculated with lesion diameters. Error bars indicate mean ± SD (Student’s *t*-test, **, *P* < 0.01; ***, *P* < 0.001).


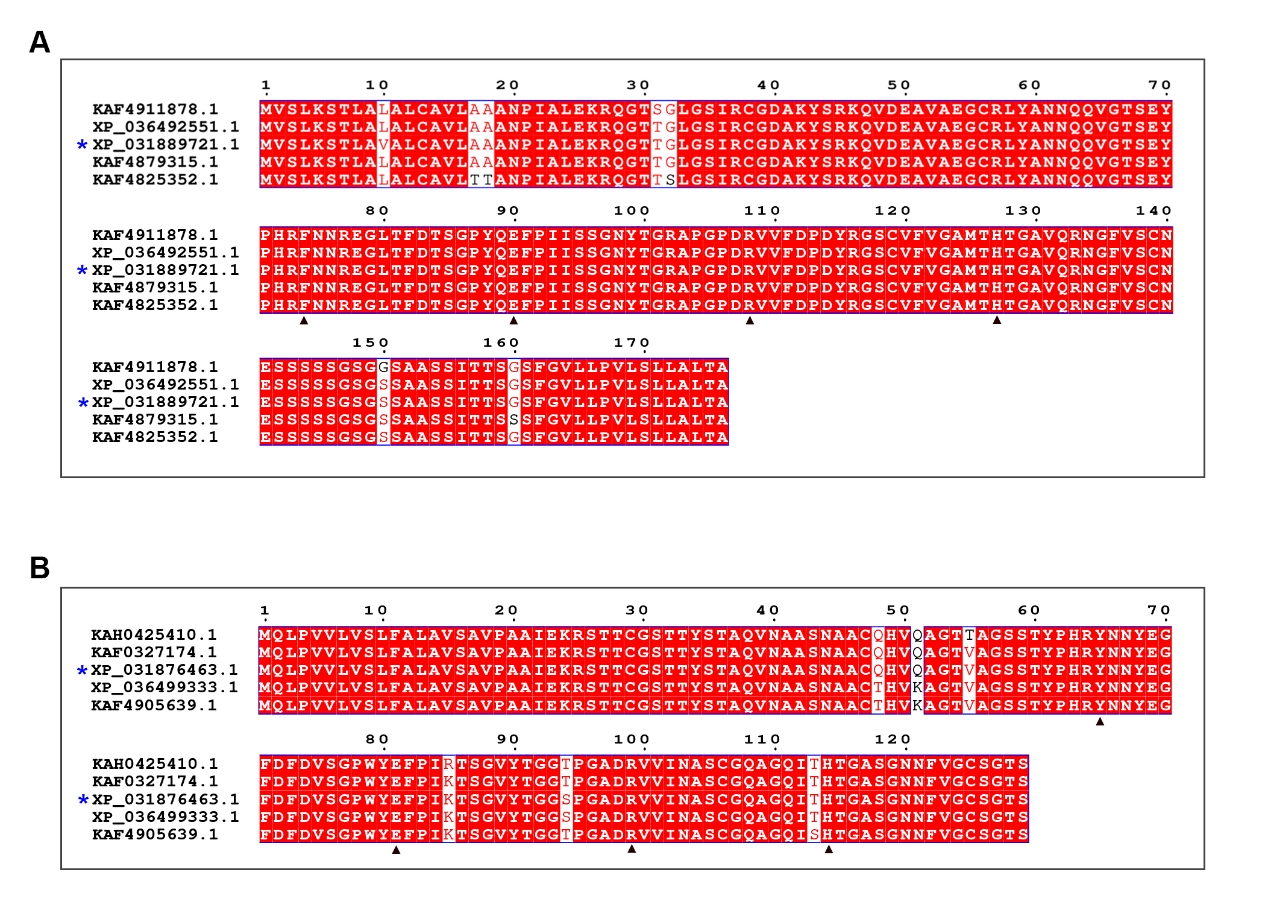


**Fig. S9** Multiple sequence alignment of CfRibo1 and CfRibo2 homologs. The alignment was performed with using ClustalW algorithm. CfRibo1 and CfRibo2 were marked with blue asterisks. Their enzymatic sites were indicated with black triangles.

**
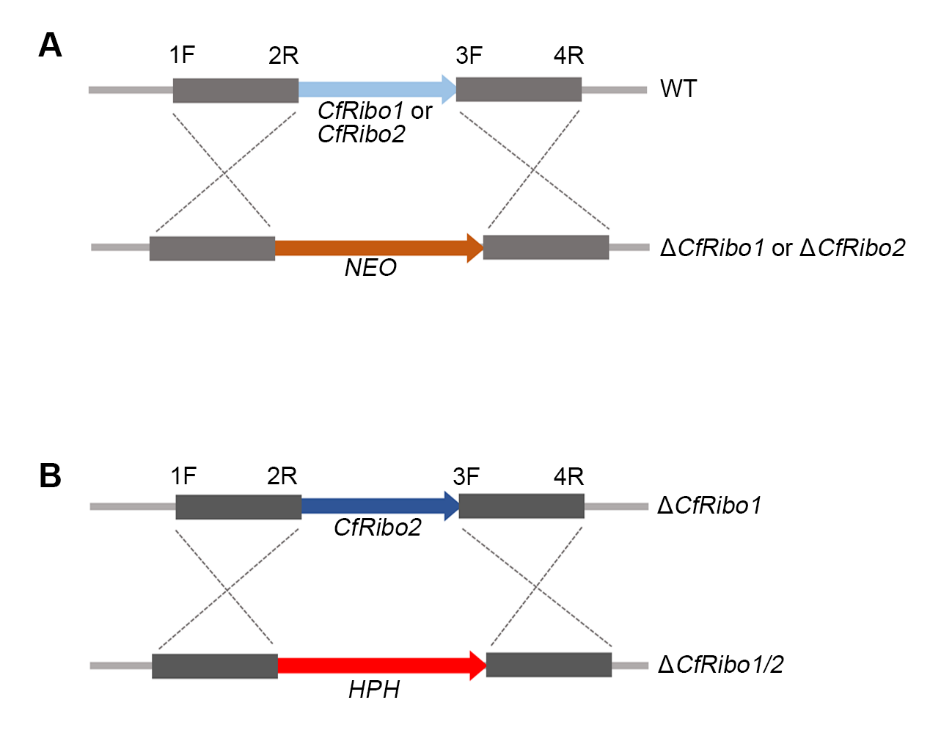
**

**Fig. S10** Schematic representation of targeted gene replacement strategy for transformations generation. *NEO* and *HPH* were used for generating resistant cassettes.
